# Bacterial diversity and successional patterns during biofilm formation on freshly exposed basalt surfaces at diffuse-flow deep-sea vents

**DOI:** 10.3389/fmicb.2015.00901

**Published:** 2015-09-10

**Authors:** Lara K. Gulmann, Stace E. Beaulieu, Timothy M. Shank, Kang Ding, William E. Seyfried, Stefan M. Sievert

**Affiliations:** ^1^Biology Department, Woods Hole Oceanographic Institution, Woods HoleMA, USA; ^2^Department of Earth Sciences, University of Minnesota, MinneapolisMN, USA

**Keywords:** hydrothermal vents, colonization, species sorting, settlement, volcanic eruption, 16S rRNA, *Epsilonproteobacteria*, disturbance

## Abstract

Many deep-sea hydrothermal vent systems are regularly impacted by volcanic eruptions, leaving fresh basalt where abundant animal and microbial communities once thrived. After an eruption, microbial biofilms are often the first visible evidence of biotic re-colonization. The present study is the first to investigate microbial colonization of newly exposed basalt surfaces in the context of vent fluid chemistry over an extended period of time (4–293 days) by deploying basalt blocks within an established diffuse-flow vent at the 9°50′ N vent field on the East Pacific Rise. Additionally, samples obtained after a recent eruption at the same vent field allowed for comparison between experimental results and those from natural microbial re-colonization. Over 9 months, the community changed from being composed almost exclusively of *Epsilonproteobacteria* to a more diverse assemblage, corresponding with a potential expansion of metabolic capabilities. The process of biofilm formation appears to generate similar surface-associated communities within and across sites by selecting for a subset of fluid-associated microbes, via species sorting. Furthermore, the high incidence of shared operational taxonomic units over time and across different vent sites suggests that the microbial communities colonizing new surfaces at diffuse-flow vent sites might follow a predictable successional pattern.

## Introduction

Microbes form the basis of the highly productive deep-sea hydrothermal vent ecosystems and serve as partners in symbiotic relationships or as food for fauna (Lutz and Kennish, 1993). These metabolically versatile chemosynthetic microbes harness chemical disequilibria created by the mixing of reduced hydrothermal fluids with oxygenated deep-sea water either above or below the seafloor (Baross and Hoffman, 1985; Jannasch and Mottl, 1985; Nakagawa and Takai, 2008). Vent fields are discrete regions separated by distances on the order of 100 km on the global mid-ocean ridge (MOR) system. Within a vent field, there are multiple sites of fluid venting, separated by meters to 100s of meters, including both focused-flow and diffuse-flow vent sites. Focused-flow sites are distinguished by characteristic black smokers, which are produced when undiluted, hot hydrothermal fluids (typically 350–400°C) exit the seafloor. In contrast, at diffuse-flow sites, seawater mixes with hydrothermal fluid within the ocean crust, and is released as warm fluids (typically < 50°C) through cracks in the seafloor.

Seafloor eruptions on intermediate- to fast-spreading MORs regularly destroy existing hydrothermal habitats, leave behind fresh basalt, and, by cracking and fissuring, create new hydrothermal emissions through openings in the basaltic seafloor (Haymon et al., 1993; Embley et al., 1995; Delaney et al., 1998; Shank et al., 1998; Huber et al., 2003; Tolstoy et al., 2006). Based on colonization experiments with artificial (non-basalt) substrates, newly exposed surfaces are colonized by microbes that form biofilms within days of exposure (Guezennec et al., 1998; Alain et al., 2004; Moussard et al., 2006). These microbial biofilms represent an important step in the primary colonization of deep-sea hydrothermal vents, as they allow microorganisms to maximize access to nutrients and energy sources in an environment typically characterized by high turbulence, and in turn support robust megafaunal communities. Initial microbial colonizers form the foundation of the vent ecosystem by playing a critical, yet poorly understood role in “conditioning” the substrate for settlement of metazoans, shaping the biological succession to follow. Microbial biofilms may facilitate the settlement and growth of particular microbial groups or act as faciliatory or inhibitory cues to metazoan larvae, thereby influencing the ultimate faunal community structure (Wieczorek and Todd, 1998; Hadfield, 2011; Wahl et al., 2012). Observational evidence suggests that larvae settle preferentially on surfaces pre-colonized by microbial biofilms (Rittschof et al., 1998; Taylor et al., 1999; Li et al., 2014). Additionally, biofilms may encourage immigration of mobile grazers (Shank et al., 1998).

**FIGURE 1:**
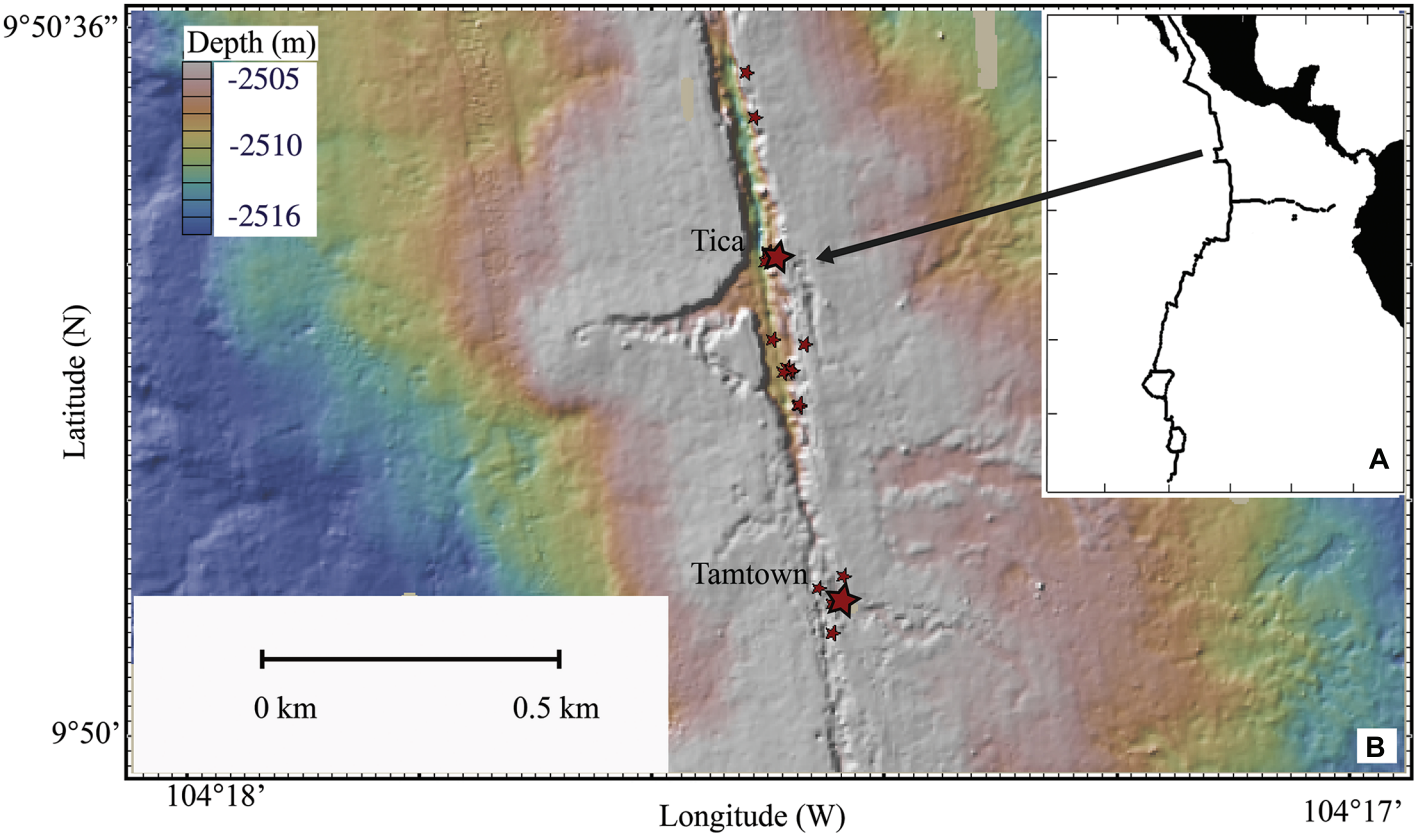
**Map of the East Pacific Rise. (A)** Location of the EPR. **(B)** Topographical map of sampling sites. Larger stars indicate Tica (site for basalt panels) and Tamtown (site for native Basalt) locations, smaller stars indicate locations of other vent sites.

Although researchers have observed biofilm formation on experimental substrates after only days (Guezennec et al., 1998; Moussard et al., 2006), little is known about the composition of microbial communities forming these early biofilms and how they change over time, as well as how biofilm communities on experimental substrates compare with those on natural substrates. Clarifying the progression of early microbial succession is key to understanding how diffuse flow vent communities establish and develop. The goal of this study is to examine how the composition of microbial communities change over time scales of a few days to 9 months on newly exposed surfaces at and adjacent to diffuse-flow venting at the 9°50′ N vent field on the East Pacific Rise (EPR; **Figure 1**). We hypothesized that the microbial communities colonizing new surfaces at diffuse-flow vents would: (1) follow a predictable successional pattern and (2) be similar among vent sites, but substantially different from non-vent habitats, as a result of species sorting from the fluid-associated source microbial community. To address these hypotheses, we compared the microbial succession both after an eruption (‘natural re-colonization’) and on basalt blocks placed within an established diffuse-flow vent community prior to the eruption (‘experimental re-colonization’).

## Materials and Methods

### Sample Collection

Basalt panels were deployed and recovered by DSV Alvin at Tica Vent on the EPR (9°50.4′ N, 104°17.5′ W: 2513-m depth; **Figure 1**) during R/V *Atlantis* research cruises AT11-04 (2003/12/01 – 2003/12/19), 11-07 (2004/02/03 – 2004/02/19), 11-10 (2004/04/07 – 2004/04/24), 11-20 (2004/11/11 – 2004/11/24). Basalt panels were deployed either at a diffuse-flow vent site (Experiment site 1: ∼15°C) or a nearby, non-vent site (Control site: ∼1.8°C). Experiment site 1 was located within a thriving patch of tubeworms (*Riftia pachyptila*) and mussels (*Bathymodiolus thermophilus*). The Control site was located 2.5-m from the experimental site, ∼½-m from mussels marking the edge of the colonized vent (Supplementary Figures S1A–C). These panels are considered ‘pre-eruption’ samples because they were collected 19–28 months before the 2005/2006 EPR eruption (Tolstoy et al., 2006; Cowen et al., 2007; Soule et al., 2007).

For the purpose of comparison with our experimental results, one post-eruption native basalt biofilm sample (hereafter referred to as ‘native Basalt’) was collected from Tamtown (9°50.3′ N, 104°17.4′ W: 2503-m depth; ∼0.58 km from Tica Vent: **Figure 1**), during the RESET06 cruise (AT15-06, 2006/06/25 – 2006/07/01). This EPR volcanic eruption was dated around the end of 2005/early 2006 (Rubin et al., 2006; Tolstoy et al., 2006; Soule et al., 2007), indicating that the Tamtown Basalt collected was ∼5–8 months-old. In addition, one biofilm sample (hereafter referred to as ‘Trap’) was collected at this location from a cylindrical stainless steel collection device placed in the outflow of an active diffuse flow vent for 4 days.

Basalt panels (10.2 cm × 10.2 cm × 2.5 cm) were constructed of basalt collected in the area of 9°N on the EPR (Supplementary Figure S1D). Before deployment, all panels were sealed in aluminum foil and autoclaved. Each panel was deployed from and recovered into an ethanol-wiped biobox mounted on Alvin’s basket, filled prior to the dive with either 0.2-μm filtered seawater or double-distilled water to prevent contamination with surface seawater (as were the native Basalt and Trap samples). All subsequent handling of the basalt panels was with sterilized gloves or tools. Control and experimental basalt panels were exposed for five time intervals: 4-, 9-, 13-, 76-days, and 9-mos [283-days (control) and 293-days (experimental)]. One panel was lost (76-days, control), leaving a total of nine panels for analysis, in addition to the native Basalt and Trap samples.

### Chemical Measurements

For additional habitat characterization, we attached a time-series temperature sensor (VEMCO) directly to one of the basalt panels at both the experimental (attached to Day 293E) and control sites (attached to Day 13C; Supplementary Figure S2) and deployed an *in situ* chemical sensor at Experiment site 1, to monitor dissolved H_2_S, Eh (oxidation-reduction potential), and temperature (Supplementary Figures S3A–C). The VEMCO temperature sensor sampled every 5 s and recorded temperatures for 293 days at Experiment site 1 and for 13 days at the control site. The chemical sensors were implanted amongst the tubeworms, using real-time temperature via an inductively coupled communication link (ICL) as a guide for placement in vent fluids (∼11°C for Experiment site 1). The chemical sensors were deployed for 13 days and sampled every 5-s. Furthermore, the chemical sensors were calibrated before and after deployment.

### Molecular Techniques

#### DNA Extraction

Environmental DNA was extracted from the basalt panel surfaces using a large-volume CTAB (hexadecyltrimethylammonium bromide) extraction. Individual basalt panels were placed into warmed (55°C) DNA extraction buffer, composed of 100 mM Tris-HCl (pH 8.0), 20 mM EDTA (pH 8.0), 1.4 M NaCl and 2% CTAB, with mercaptoethanol added to 0.2% via syringe filter. Filter-sterilized Proteinase K solution and sodium dodecyl sulfate (SDS) solution were added to final concentrations of 0.1 mg/mL and 0.65% respectively. Covered by this extraction solution, each block was agitated on a shaker table for 2 h at 55°C. Biofilm removal was confirmed by inspection of the extracted blocks under a dissecting microscope. DNA was extracted from this solution with an equivalent volume of phenol:chloroform:isoamyl alcohol (25:24:1, pH 8.0), followed by an equivalent volume of chloroform:isoamyl alcohol (24:1). To precipitate the DNA, 0.1 volume of 3 M sodium acetate and 2.5 volumes of cold 100% ethanol were added, then placed into -20°C for 3 h before centrifuging at 10,000 × *g* for 15 min. After decanting the supernatant, the pellets were covered with cold 70% ethanol and centrifuged at 16,000 × *g* for 5 min. The ethanol was pipetted off and the pellets were dried. Isolated DNA was resuspended in 50–200 μl sterile water and kept at -80°C until use. Environmental DNA from Trap and native Basalt samples was extracted with using the Ultraclean Soil DNA extraction kit (MoBio Laboratories).

#### Clone Library Construction and Full-Length 16S rRNA Sequencing

The 16S rRNA region of environmental DNA was amplified (27F, 1492R primers) and replicates of 10 PCR amplifications (15 cycles each) were combined, precipitated using a QIAquick PCR purification kit (Qiagen), resuspended in 35 μl of sterile water and purified using the QIAquick gel extraction kit (Qiagen). Replicate PCR reactions were combined in order to minimize PCR bias (Polz and Cavanaugh, 1998) and only 15 cycles were used to decrease the formation of chimeric sequences and Taq error. The combined products were then reamplified with five additional PCR cycles to minimize the formation of heteroduplex molecules (Thompson et al., 2002) and purified using a QIAquick gel extraction kit (Qiagen).

Purified PCR products of the 16S rRNA gene were cloned with the Strataclone PCR cloning kit (Stratagene) for sequencing. Nearly complete, double-stranded sequences of 16S rRNA genes (∼1,475 bp) were sequenced on a 96-capillary 3730xl DNA analyzer (Applied Biosystems) with primers M13F and M13R. For all libraries, single strand sequences were grouped by 97% similarity with the program Sequencher (version 4.1.2, Applied Biosystems) and one representative of each group was selected to sequence both forward and reverse strands. These groupings were designated operational taxonomic units (OTUs). Sequences were tested for the presence of chimeras with the program Mallard (Ashelford et al., 2006) and with the Bellerophon server (Huber et al., 2004). The full-length 16S rRNA sequences reported in this paper have been deposited in the GenBank database under accession numbers KT257735-KT257859.

#### 454 Pyrosequencing of V4 16S rRNA Tags

Primers 517F and 806R were used to target the V4 region of the 16S rRNA gene (Caporaso et al., 2010). For multiplex sequencing, eight forward primers were synthesized (Eurofin), each with a different tag. PCR reactions (50 μl volume) contained 250 nM of each of forward and reverse primers, 5 ng of template DNA and were performed using Picomaxx taq polymerase (Invitrogen, Carlsbad, CA, USA) using the thermal profile 95°C for 2 min followed by 25 cycles of denaturation at 94°C for 15 s, primer annealing at 61°C and extension at 72°C for 45 s, with final extension of 72°C for 3 min. Amplicons were sequenced at EnGenCore, now Selah Genomics, using 454 FLX chemistry. Raw sequences were processed with QIIME 1.8.0 (Caporaso et al., 2010). Sequences were excluded from analysis if they had a mean quality score <25, were either <200 or >500 bp in length, contained ambiguous nucleotides or had any mismatches in the forward and reverse primers. The sequences were assigned to individual samples by their adapter tags and the 16S rRNA primers were removed prior to analysis. The resulting data sets contained 254 bp of the bacterial V4 region. Potential chimeras were identified and removed using ChimeraSlayer. Trimmed sequences were then classified with RDP classifier. OTUs were clustered at 97% similarity based on a distance matrix generated with the program QIIME. SFF files were assigned GenBank SRA Bioproject number PRJNA288972.

#### Phylogenetic and Statistical Analysis

Phylogenetic trees were constructed from aligned clone sequences and closely related environmental clones and cultures using the neighbor-joining algorithm in the MEGA6 software package (Tamura et al., 2013) and the Silva release 119 database. Tag sequences that were identical to a clone sequence were identified in trees. Bootstrap values were calculated using MEGA6. Alpha and beta diversity estimates were calculated using QIIME. After trimming each sample to an equal number of tags, bacterial diversity was estimated with N_obs_ (observed richness), Chao1 (non-parametric richness estimator), non-parametric (np) Shannon diversity index, Simpson Evenness and Equitability. Structure of the pyrosequenced microbial communities was compared with two different methods. Principal coordinate analysis (PCoA) maps the samples on a set of orthogonal axes in order to explain the maximum amount of variation by the first coordinate and the second largest amount of variation by the second coordinate based on weighted UniFrac values (Lozupone et al., 2010). The UPGMA (unweighted pair group method with arithmetic mean) distance tree is based on a hierarchical clustering in which topological relationships are identified in order of similarity. The robustness of the UPGMA clusters was tested with jackknife analysis, based on 100 randomized subsamples.

## Results

### Habitat Chemistry

Based on the VEMCO temperature sensors, temperature at Experiment site 1 ranged from 2.6 to 21.4°C (average 10.7°C) over the course of the experiment, while temperatures at the Control site ranged from 1.8 to 3.0°C (average 2.3°C) over the first 13 days of the experiment (Supplementary Figure S2). The temperature recorded by the *in situ* chemical sensor (Supplementary Figure S3A), ranged from 8.7 to 20.9°C (average 17.2°C), but only measured for the first 13 days of the experiment. Immediately following deployment, temperature increased from ∼9 to 20°C, but then subsequently decreased and increased in a pattern suggestive of tidal influences (Supplementary Figure S3A). Total dissolved sulfide and Eh (Supplementary Figures S3B,C) were initially at or below detection, but increased, or became more reducing subsequently. After ∼1–2 days, data reveal steady state redox conditions with small-scale changes that likely reflect temperature variability, although the cause of this is difficult to quantify unambiguously. In general, the dissolved sulfide concentrations ranged from 10 to 100 μM and the mean value of dissolved sulfide was in the range of 20–30 μM.

### Phylogenetic Diversity

**FIGURE 2:**
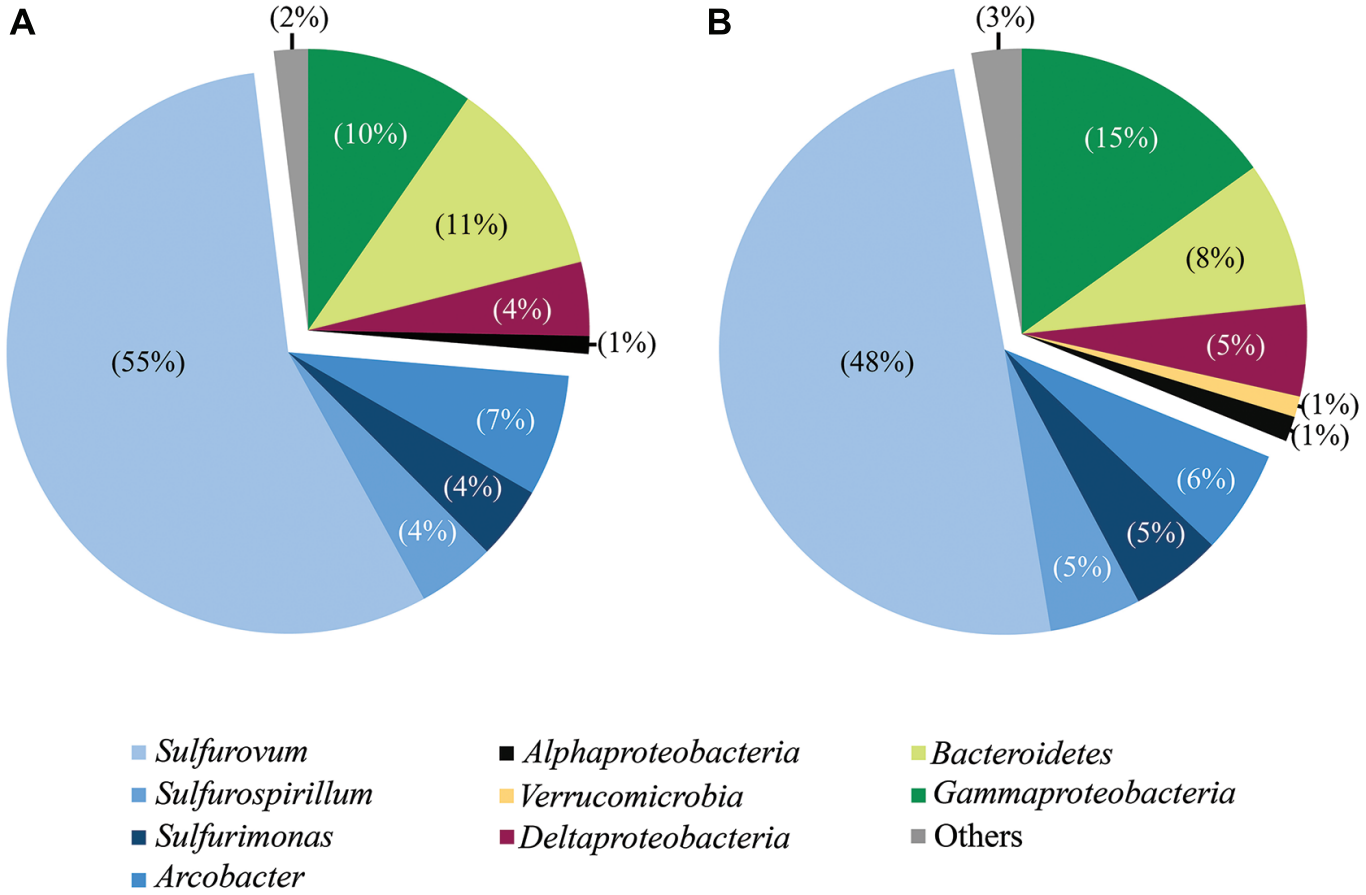
**Taxonomic breakdown and relative abundance for combined tag **(A)** and clone **(B)** sequences.** All libraries analyzed by both methodologies are grouped (all libraries except Trap and day 4E).

**FIGURE 3:**
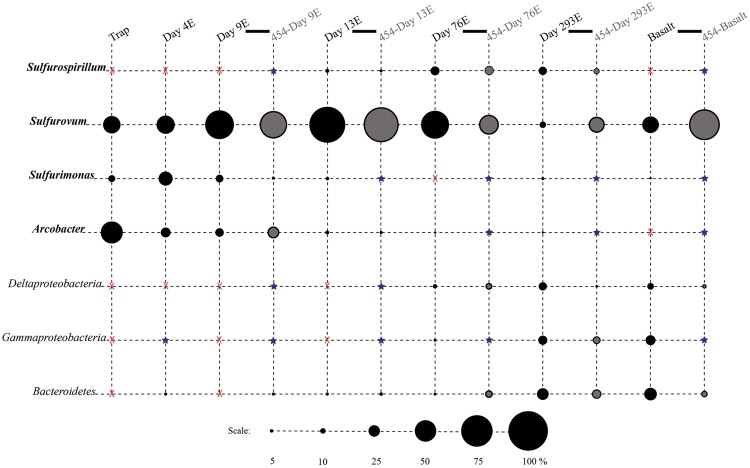
**Relative proportions of abundant phylogenetic groups for experimental treatments and Basalt.** Pyrosequenced samples are labeled as 454-sample name (gray shading). All other samples were sequenced with the Sanger method (black shading). Epsilonproteobacterial groups are labeled in bold italics. Groups in which no clones/tags were sequenced are indicated with a red ‘X.’ Groups which were detected at or below 2% are indicated with a blue star.

**FIGURE 4:**
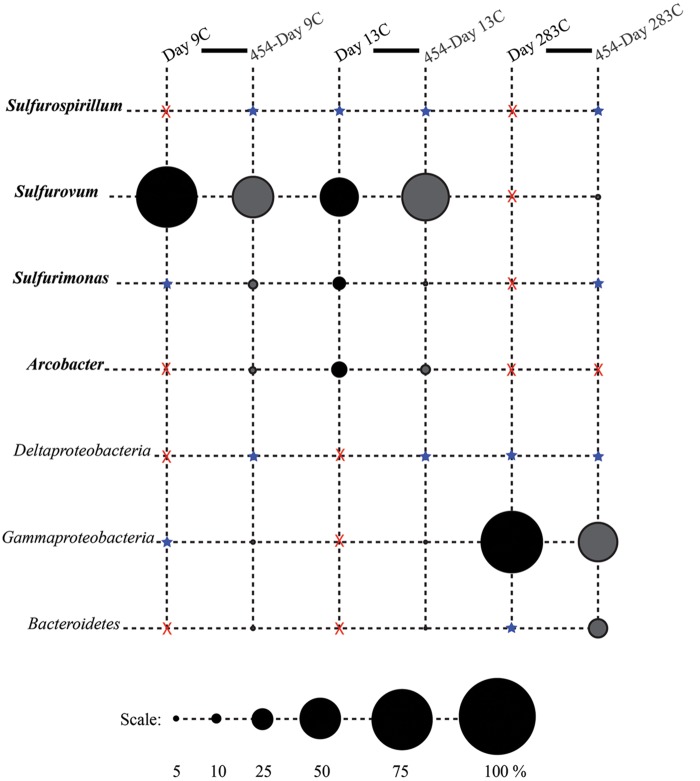
**Relative proportions of abundant phylogenetic groups for control treatments.** Pyrosequenced samples are labeled as 454-sample name (gray shading). All other samples were sequenced with the Sanger method (black shading). Epsilonproteobacterial groups are labeled in bold italics. Groups in which no clones/tags were sequenced are indicated with a red ‘X.’ Groups which were detected at or below 2% are indicated with a blue star.

We studied bacterial diversity on basalt surfaces at diffuse flow vent sites (**Figure 1**) using a combination of full-length Sanger sequencing of 16S rRNA clones (full-length clones) and 454-pyrosequencing of the hypervariable V4 region of 16S rRNA (V4 tags). While the use of V4 tags allowed a more extensive assessment of microbial diversity of the samples, the generation of full-length sequences provides a means to perform phylogenetic analyses and to verify the taxonomic assignments of the most dominant groups. Sample 76C was not recovered and sample 4C had insufficient DNA for molecular analysis. Using the same DNA extracts, we amplified the V4 region of the 16S rRNA gene for pyrosequencing of eight samples (all samples amplified for clone libraries except day 4E and Trap), generating over 60,500 reads, with 23,155 reads passing through the quality control criteria. Both methods (Sanger sequencing and 454-pyrosequencing) detected the same overall pattern of community composition (**Figure 2**) and changes with time (**Figures 3** and **4**). Similarly, other environmental studies of hydrothermal vent habitats have indicated a good correspondence between clone libraries and tag pyrosequencing approaches (Sylvan et al., 2012b; Forget and Juniper, 2013). Multiple attempts to amplify archaeal 16S rRNA using archaeal specific primers were unsuccessful. Although other studies have recovered archaeal DNA from high temperature vents (Byrne et al., 2009; Opatkiewicz et al., 2009), other researchers have also failed to amplify Archaea from lower temperature diffuse flow vents (Forget et al., 2010; Forget and Juniper, 2013).

**FIGURE 5:**
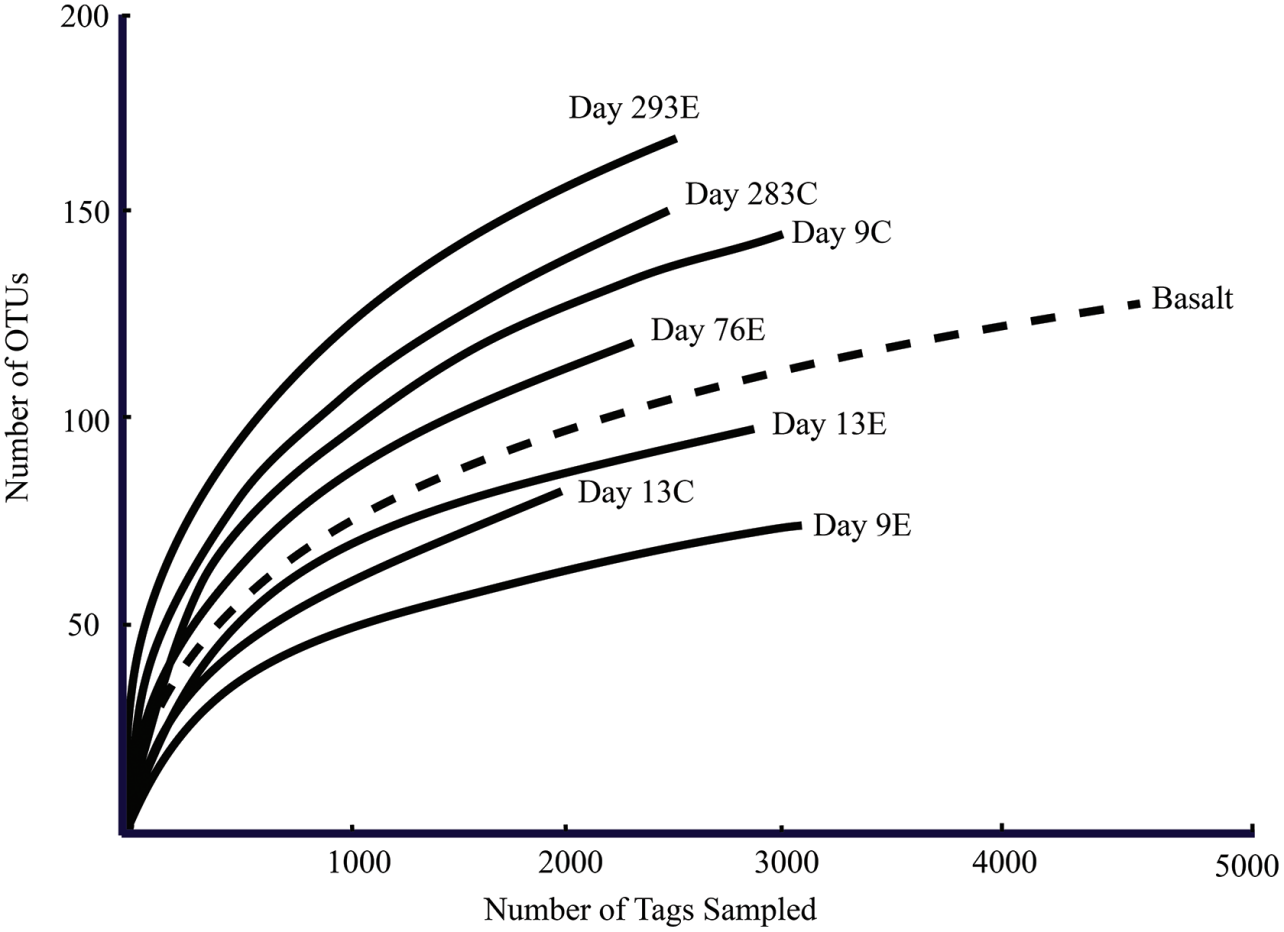
**Rarefaction curves for panel samples (Tica vent – solid line) and Basalt sample (Tamtown vent – dashed line).** OTUs were calculated with a cutoff of 3%.

According to rarefaction data and diversity indices based on the 454 tag sequences (**Figure 5**; **Table 1A**), the 9-months experimental panel hosted the most diverse microbial community. The 9-months control has a microbial community only slightly less diverse than the corresponding experimental time point, and comprised the largest number of singletons (62), as well as the second highest calculations for Chao1 and Shannon indices (based on 454 tag sequence analysis). In general, the early time points (<2 weeks exposure, both control and experimental) had the least diverse communities and diversity increased with age. However day 9C was an exception, with its relatively large number of singletons (58) and relatively high Chao1 value (144). The estimated diversity of the native Basalt sample falls in between early and late time-points, as confirmed with the Shannon index, which incorporates both the richness and evenness of the community. The calculated Simpson Evenness and Shannon indices both indicate that the experimental communities become more even as they age (**Table 1A**). In comparing the two methods, both libraries found 293E to be the most diverse sample, and both found 9E to be the least diverse sample. Interestingly, the tag library revealed greater diversity of two control samples, 283C and 9C (**Tables 1A,B**).

**Table 1A:** Diversity estimates from 16S rRNA amplicon libraries: 454 tag sequences.

Sample	Reads	N_obs_	Singletons	Chao1^∗^	Shannon	Simpson evenness	Equitability
Day 9E	3148	74	24	109 (86–170)	3.21	0.017	0.52
Day 13E	2892	98	30	123 (107–160)	3.96	0.012	0.60
Day 76E	2309	118	45	201 (155–301)	4.49	0.009	0.65
Day 293E	2568	168	54	230 (199–292)	5.31	0.006	0.72
Basalt	4732	132	43	188 (158–255)	3.69	0.010	0.52
Day 9C	2993	144	58	216 (181–285)	4.05	0.008	0.56
Day 13C	2003	82	39	156 (114–256)	3.31	0.015	0.52
Day 283C	2510	151	62	224 (189–290)	4.63	0.007	0.64

*^∗^The ranges shown are 95% confidence intervals.*

**Table 1B:** Diversity estimates from 16S rRNA amplicon libraries: clone sequences.

Sample	Clones	N_obs_	Singletons	Chao1^∗^	Shannon	Simpson evenness	Equitability
Trap	63	13	6	20 (14–55)	2.52	0.113	0.70
Day 4E	55	20	9	26 (21–47)	3.75	0.056	0.87
Day 9E	56	13	7	24 (15–67)	2.91	0.094	0.79
Day 13E	50	17	8	26 (19–62)	5.10	0.066	0.88
Day 76E	59	25	15	60 (35–151)	3.59	0.044	0.87
Day 293E	53	38	27	82 (54–155)	4.05	0.027	0.97
Basalt	64	27	19	70 (40–166)	3.24	0.041	0.83
Day 9C	51	12	7	17 (13–40)	2.97	0.154	0.56
Day 13C	46	15	8	24 (17–60)	3.97	0.079	0.83
Day 283C	57	15	9	27 (18–69)	2.01	0.082	0.76

*^∗^The ranges shown are 95% confidence intervals.*

### Comparison of Bacterial Communities

#### Composition of Libraries

Phylotypes from three subdivisions of *Proteobacteria*, i.e., *Gamma*-, *Epsilon*-, and *Deltaproteobacteria*, as well as *Bacteroidetes* constituted the majority of sequences in all samples (**Figures 2A,B, 3**, and **4**). The overall results indicate a shift, over a period of months, from a community dominated by *Epsilonproteobacteria*, to a more diverse community including *Bacteroidetes, Gamma*-, and *Deltaproteobacteria*, among others (**Figures 3** and **4**).

**FIGURE 6:**
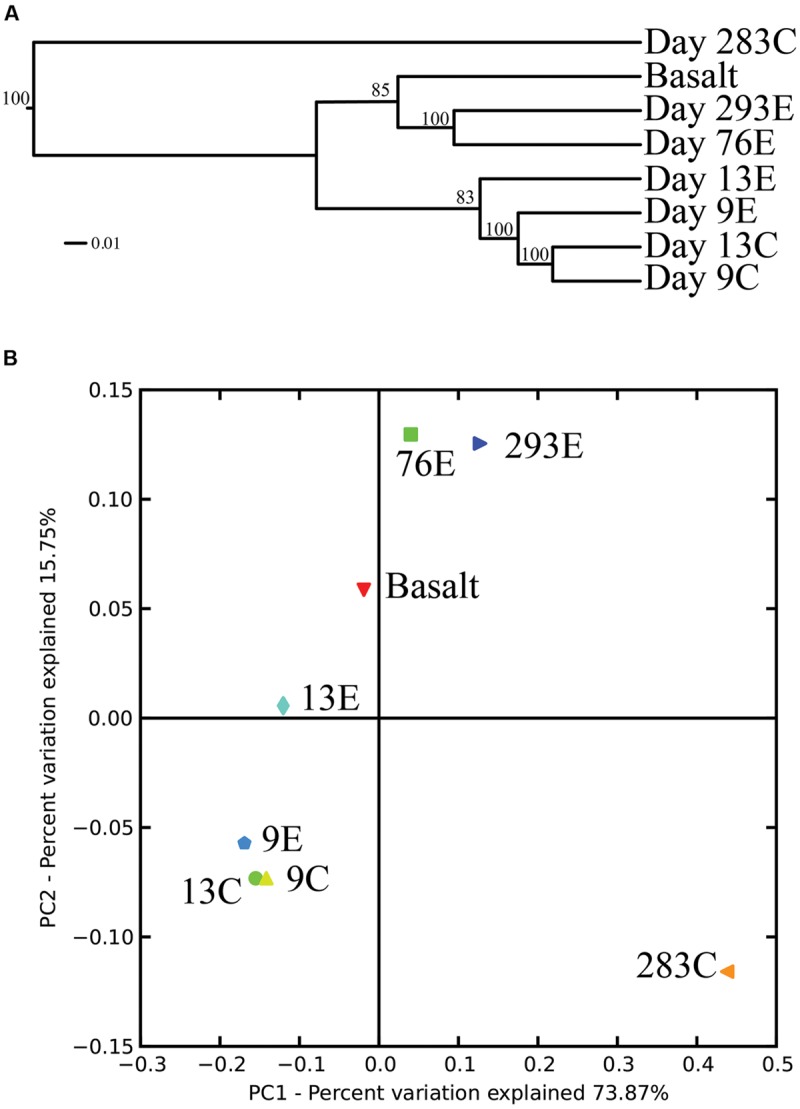
**(A)** Unweighted pair group method with arithmetic mean (UPGMA) distance tree of the 454 bacterial community structure. Jacknife support is indicated at the nodes. **(B)** PCoA plots of all 454 libraries with weighted UniFrac. The first two principal coordinate axes in PCoA and percentages of variation that they explain are shown.

Surprisingly, the composition of the control and experimental libraries were highly similar for the first 13 days, and only diverged sometime thereafter. In particular, days 9E, 9C, and 13C group in their own branch of the UPGMA tree and form a cluster in the weighted PCoA plot (**Figures 6A,B**). Libraries from days 76E and 293E formed a separate group, while bacterial communities found on the native Basalt and 13E shared similarities with both of these clusters. Both measures indicate that the bacterial community found associated with panel 283C is the most disparate from all other libraries (**Figures 6A,B**).

Among the *Epsilonproteobacteria*, >90% of tag OTUs were detected in multiple panel samples. Of the total 132 OTUs identified for the native Basalt sample, only seven OTUs were not present in the experimental panels and these OTUs comprise <2% of the total tags for the native Basalt sample (Supplementary Tables S1A–D and 2A–D). Within the *Epsilonproteobacteria*, 94% of the OTUs detected for the native Basalt sample were also found on the experimental panels, 0.58 km apart [Tamtown (native Basalt) and Tica vent (panel samples)]. From the earliest timepoints, >38% of the *Epsilonproteobacterial* OTUs found at day 4E (Tica vent) were also detected in the Trap library (Tamtown; Supplementary Table S2A).

#### Epsilonproteobacteria

**FIGURE 7:**
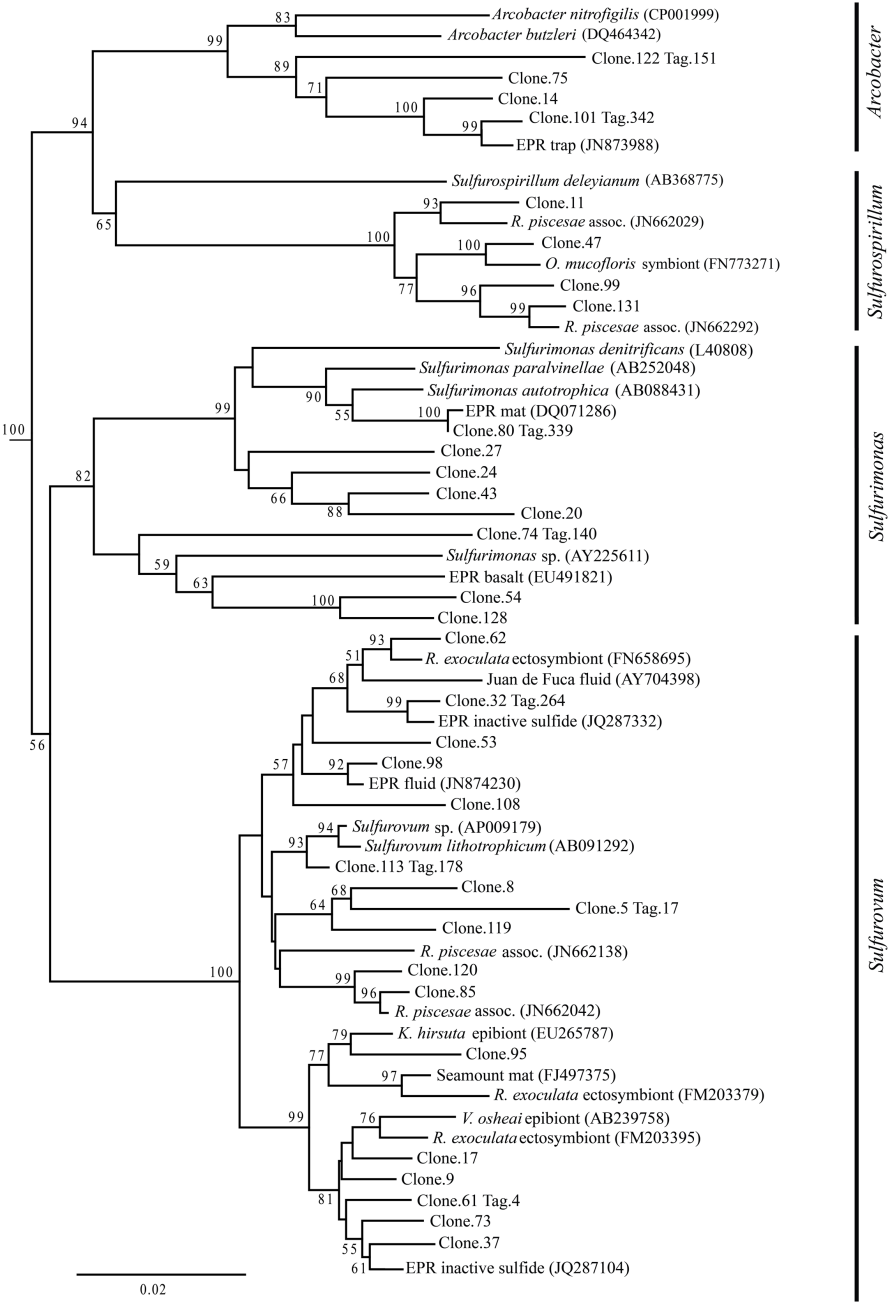
***Epsilonproteobacteria* phylogenetic tree.** Neighbor-joining trees were constructed from aligned, full-length clone 16S rRNA sequences of OTUs detected more than once, based on 1,197 homologous positions filtered by base frequency (50%) using MEGA6. Tag sequences that were identical to a clone sequence were identified. Bootstrap values were calculated in MEGA6, and nodes with >50% support, based on 1,000 replicates are displayed. *Hydrogenobacter hydrogenophilus* (Z30242) and *Thermosulfidicus takaii* (AB282756) were used as outgroups.

Up to day 76, *Epsilonproteobacteria* accounted for the majority of tags and clones in all libraries (65–95% tags; 82–96% clones). The Trap library was exclusively comprised of *Epsilonproteobacteria* and every time point for the first 2 weeks contained >90% *Epsilonproteobacterial* clones and tags. In particular, the genus *Arcobacter* was prevalent until day 13, comprising up to 25% of all tags (day 9E) and >50% of all clones (Trap; **Figure 3**). But after 2 weeks, the proportion of *Arcobacter* decreased and never comprised more than 2.5% of the total tags or clones. No *Arcobacter* clones/tags were found for 283C. For the native Basalt sample, no *Arcobacter* clones were detected and few 454 tags were recovered (20 tags, <0.1%). Even though *Arcobacter* comprise 7 and 6% of all tags and clones, and over a quarter of all tag sequences for day 9E, these sequences were all grouped within only a few OTUs. Only seven *Arcobacter* tag OTUs were detected and over 84% of all *Arcobacter* tags belonged to a single OTU (Supplementary Table S1A). This OTU (Clone.101:Tag.342) shares 99% sequence identity with a clone (JN873988) collected from a Trap 30 m southwest of vent Bio9 at the EPR, which collected hydrothermal plume particles (**Figure 7**; Supplementary Figure S4A; Sylvan et al., 2012a).

Within the *Epsilonproteobacteria*, OTUs belonging to the *Sulfurovum* genus constituted the dominant fraction of tags and clones across all time points except the 9-months experimental and control (293E, 283C) and Trap sample (**Figures 3** and **4**). Many *Sulfurovum*-related tags/clones were most closely related to sequences previously identified from hydrothermal vent environments, and in particular to clones found at vent sites also colonized by vent fauna (Huber et al., 2006; Goffredi et al., 2008; Sylvan et al., 2012b; Forget and Juniper, 2013; **Figure 7**). The most abundant sequence (Clone.32 Tag.264) comprised 16% of all tag sequences and 19% of all clone sequences and is >99% similar to a *Sulfurovum*-like sequence from an extinct sulfide (JQ287332; Sylvan et al., 2012b; Supplementary Figure S4A). Many of the identified tags/clones were only distantly related to any cultured representatives.

*Sulfurimonas*-related tags were also more common in early time points, comprising between 2 and 5% of all tags for days 9 and 13 (**Figures 3** and **4**). *Sulfurimonas* tags make up less than 1% of all tags for 293E (0.9%) and 283C (0.4%) and only 1.4% of all native Basalt tags. In clone libraries, *Sulfurimonas*-related sequences accounted for 27% of all clones of the earliest time point (day 4E) and subsequently decreased. When samples were grouped (all except Trap and day 4E), *Sulfurimonas*-related sequences comprised 5% of the total for clone libraries and 4% of the total for 454 tag libraries (**Figures 2A,B**). One OTU (Clone.80 Tag.339) was closely related to *Sulfurimonas paralvinellae* (>97% similarity; **Figure 7**), a mesophilic, facultatively anaerobic, strictly chemolithoautotrophic epsilonproteobacterium using the oxidation of H_2_ or reduced sulfur compounds as an energy source (Takai et al., 2006).

*Sulfurospirillum*-related tags were first detected in low abundances at day 9 (9C and 9E < 1% total; **Figures 3** and **4**). Relative abundances peaked on day 76E, where they comprised 19% of all tags for that sample, and were also found at day 293E (321 tags, 12% total). Clones related to *Sulfurospirillum* species were not detected until day 13. Initially, clones belonging to *Sulfurospirillum* comprised only 4% of the library for 13E, but made up 17% of all clones by time points 76E and 293E, respectively. Up to this point all described species of the genus *Sulfurospirillum* are heterotrophs capable of using sulfur as an electron acceptor (Campbell et al., 2006).

#### *Gamma-* and *Deltaproteobacteria* and *Bacteroidetes*

**FIGURE 8:**
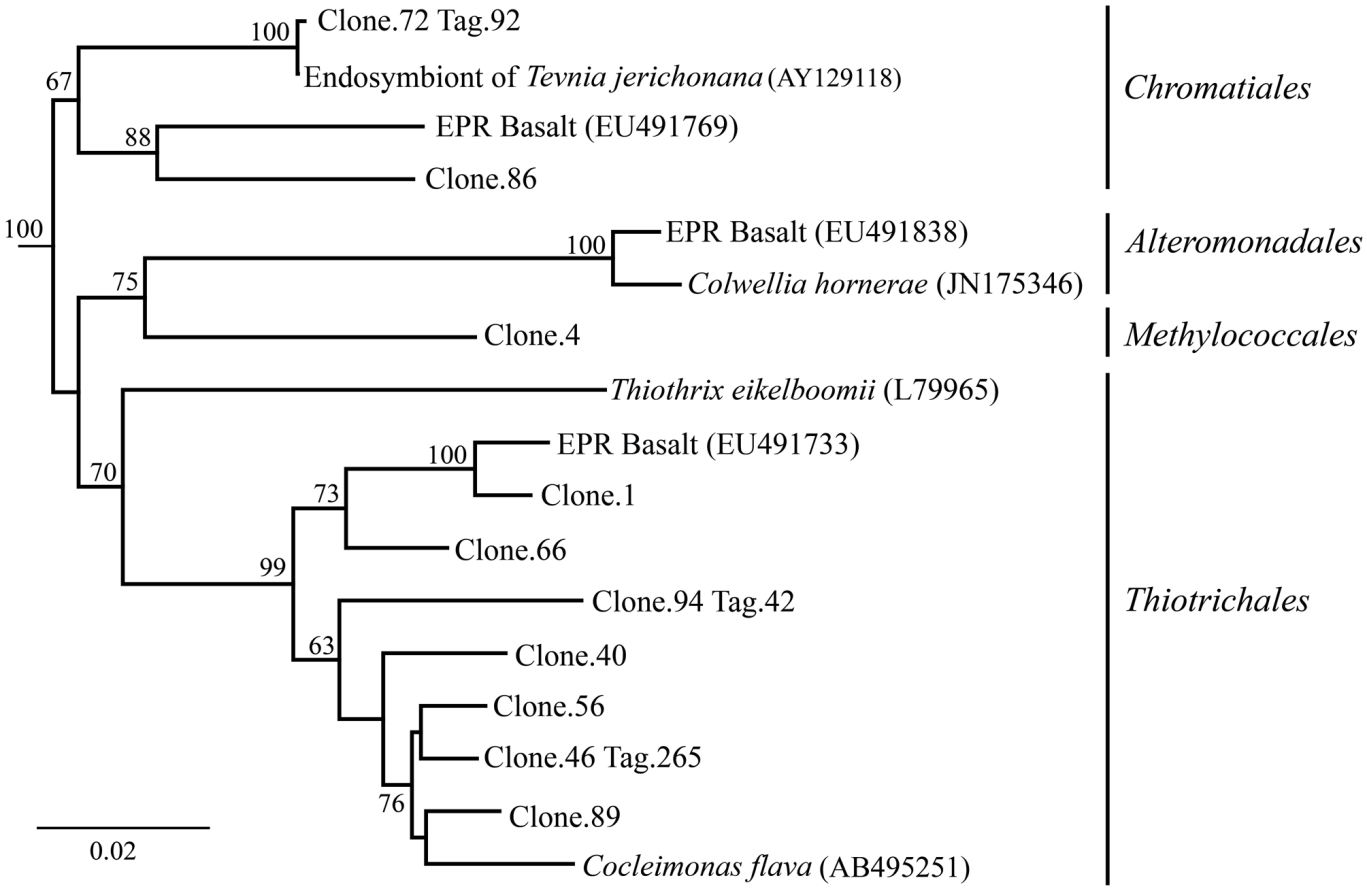
***Gammaproteobacteria* phylogenetic tree.** Neighbor-joining trees were constructed from aligned, full-length clone 16S rRNA sequences of OTUs detected more than once, based on 1,153 homologous positions filtered by base frequency (50%) using MEGA6. Tag sequences that were identical to a clone sequence were identified. Bootstrap values were calculated in MEGA6, and nodes with >50% support, based on 1,000 replicates are displayed. *Haliangium tepidum* (NR_024781) and *Kofleria flava* (NR_041981) were used as outgroups.

After 9 months of exposure, the experimental panel library comprised a mixture of *Bacteroidetes, Epsilon-* and *Gammaproteobacteria*, and the control library was dominated by *Gammaproteobacteria* (>57% of all tags and >90% of all clones in the control library (**Figure 4**). Pyrosequencing results recovered a high proportion of *Thiotrichales* tags (42%) distributed among 13 OTUs. Within the *Thiotrichales*, Clone.1 is >97% similar to clones previously identified on an off-axis EPR basalt, and implicated in basalt alteration reactions (GenBank accession number EU491733; **Figure 8**; Santelli et al., 2009). Interestingly, only one *Chromatiales* OTU tag was detected, but it constituted 12% of all gammaproteobacterial tags and is particularly abundant in the 9-months experimental samples (9% of all 293E tags). This OTU (Clone.72 Tag.92) shares 99% sequence identity with the endosymbiont of *Tevnia jerichonana* (**Figure 8**; Supplementary Figure S4B). The typical deep-sea psychrophilic and barophilic genus *Colwellia* was detected in low abundance (<3%) in all tag samples except the native Basalt, 76E and 293E (Supplementary Figure S4B; Supplementary Table S1B).

*Bacteroidetes* were found in all pyrosequencing libraries, and comprised ∼1-quarter of all tags for both the 9-months experimental and control panels (**Figures 3** and **4**). Most tag sequences were related to the *Flavobacteriales*, an order that, based on cultures, is comprised of heterotrophic bacteria that have a range of physiologies and are found in diverse ecological niches (Kirchman, 2002). Pyrosequencing libraries comprised a relatively high number of *Bacteroidetes* OTUs (108 OTUs across all samples), but most were detected at low abundance (i.e., only seven OTUs with >50 tags/sample).

Until day 76, *Deltaproteobacteria* were not detected by clone libraries and comprised <1% of the tag sequences (**Figures 3** and **4**). At 293 days, 5% of the tags on the experimental panel belonged to the *Deltaproteobacteria*, but higher proportions were found on the native Basalt (9%) and day 76E (14%) panel. Over half the *Deltaproteobacteria* clones (59%) and tags (71%) sequenced belonged to the order *Desulfobacterales*, and most of these belonged to the sulfur-disproportionating genus *Desulfocapsa*. For individual samples, the greatest proportion of *Desulfocapsa* tags were detected on native Basalt (8%) and day 76E (9%; Supplementary Table S1D).

## Discussion

### Biofilm Formation and Microbial Succession

From our results it is clear that diffuse-flow vent microbial communities evolve over short time scales (from a few days to 9 months) from being composed almost exclusively of *Epsilonproteobacteria* (namely, *Sulfurovum* and *Arcobacter*) to a more diverse assemblage of *Epsilon*-, *Gamma-, Deltaproteobacteria* and *Bacteroidetes*. In contrast, sites outside of the diffuse venting region developed into a community dominated by *Gammaproteobacteria* and *Bacteroidetes*, albeit of similarly high diversity. Surprisingly, the compositions of the microbial communities on the control and experimental basalt panels were highly similar for the first 13 days, and only diverged sometime thereafter. This is despite the fact that the temperature did not show a hydrothermal signal at the Control site. Fluid-associated organisms were likely transported away from the vent site by currents, providing a constant means for microbes to be delivered to the Control site, only 2.5 m away from the experimental site. While chemoautotrophic vent bacteria were able to settle on the control panels, they were probably not able to grow to appreciable numbers, preventing the formation of extensive biofilms. In addition to visual observations, this was apparent in the much lower DNA yield from the control panels compared to the experimental panels, such that the earliest control time point (day 4C) did not have sufficient DNA for PCR amplification. Over time, bacteria present in the ambient deep-sea water (i.e., *Alteromonadales*) as well as bacteria able to extract energy from fluid-associated minerals or organic matter (Templeton et al., 2009) or from the basalt itself (Santelli et al., 2008, 2009), colonized the control panels, resulting in a community distinct from the experimental basalt surfaces. For the purposes of this paper, the 9-months time points allow a comparison between experimental and control settings, while the early libraries allow insights into which bacterial groups are early colonists on freshly exposed basalt surfaces in proximity to diffuse-flow venting.

The dominance of *Epsilonproteobacteria* on surface basalt biofilms within the diffuse-flow region is consistent with previous observations showing that this group is commonly found at deep-sea hydrothermal vents, particularly in diffuse-flow vent fluids, microbial mats, and in association with macrofauna. As a group, *Epsilonproteobacteria* demonstrate a diverse range of physiologies. They are capable of oxidizing reduced sulfur compounds or hydrogen autotrophically or mixotrophically and can use oxygen, nitrate, or sulfur as electron acceptors.

They are believed to have important roles in the cycling of sulfur, nitrogen, and hydrogen at deep-sea hydrothermal vents (Longnecker and Reysenbach, 2001; Nakagawa et al., 2005; Takai et al., 2005; Sievert and Vetriani, 2012). In part, this broad range of metabolic activities allows *Epsilonproteobacteria* to colonize and proliferate in numerous vent environments (Campbell et al., 2006, 2013). Based on known metabolic activities of cultured representatives, it would appear that the earliest colonists (*Arcobacter, Sulfurovum, Sulfurimonas*) are possible chemolithoautotrophs that might be reducing nitrate and/or oxygen and oxidizing reduced sulfur compounds and/or hydrogen (Campbell et al., 2006). Certain bacterial sequences (Clone.113 Tag.178) are closely related (>98% similar) to a known sulfur-oxidizing chemolithoautotroph, *Sulfurovum lithotrophicum* (Inagaki et al., 2004). These putative autotrophic *Epsilonproteobacteria* are likely supplied in abundance from diffuse-flow vent fluids, both as particle-associated and free-living populations originating in the sub-seafloor mixing zone of hydrothermal fluid and seawater (Huber et al., 2003, 2007; Opatkiewicz et al., 2009; Sylvan et al., 2012a; Campbell et al., 2013). Autotrophic taxa capable of forming biofilms could settle out from vent fluids and rapidly colonize exposed surfaces. In this study, biofilms were visibly detected after only 4 days at both Tica (experimental panels) and Tamtown, indicating the rapid rate at which these microorganisms can establish high population densities.

Based on our full-length clone and V4 tag data, initial biofilms consisted of *Arcobacter* and *Sulfurovum*-like organisms. Other researchers have documented biofilms formed by filamentous-sulfur producing bacteria belonging to the genus *Arcobacter* at different vent locations after only a few days (Taylor and Wirsen, 1997; Taylor et al., 1999; Wirsen et al., 2002; Moussard et al., 2006). *Arcobacter* species seem to grow optimally and selectively at high sulfide concentrations (Wirsen et al., 2002; Moussard et al., 2006; Sievert et al., 2007). Based on the pattern observed in this study and by others (Taylor et al., 1999; Moussard et al., 2006; Sievert et al., 2007, 2008), we propose that this genus specializes in rapid colonization (<4 days), quickly utilizing available sulfide (Sievert et al., 2007). It is also possible that elemental sulfur formed by *Arcobacter* (Wirsen et al., 2002) serves as the substrate for other sulfur-oxidizing bacteria, such as *Sulfurovum* and *Sulfurimonas* (Pjevac et al., 2014). After ∼2 weeks, *Arcobacter* species declined and were replaced, and possibly outcompeted, by *Sulfurovum* and other epsilonproteobacterial species with potentially more diverse metabolisms and/or different life strategies. The relatively high micro-diversity observed among *Sulfurovum*-related OTUs (31 OTUs detected, clone and tag libraries) contrasts with the low diversity observed among the *Arcobacter*-related OTUs (seven OTUs detected, clone and tag libraries). Possibly, these co-inhabiting *Sulfurovum* phylotypes might be adapted to subtle differences in environmental parameters such as temperature or sulfide concentrations, and therefore able to fill multiple metabolic niches (Sievert and Vetriani, 2012). As a result, the elevated micro-diversity among *Sulfurovum*-like organisms might translate to greater metabolic diversity and greater competitive ability, leading to the decline of arcobacters.

Over time, the community dominated by putative chemoautotrophs changes to a community with an increased proportion of microbial groups with known heterotrophic representatives, i.e., *Bacteroidetes, Delta*- and *Gammaproteobacteria* and *Sulfurospirillum*. This increase in bacterial diversity with biofilm age is reflected in the Chao1 richness indices and rarefaction curves, and can be seen for both experimental and control locations. With the exception of 9C, the communities found on the youngest samples are characterized as being the least diverse and most uneven, while the opposite was true for the 9-months samples. Most likely, the initial colonization and growth by vent fluid-associated chemoautotrophs leads to an increased availability of organic matter and the establishment of complex, three-dimensional biofilm structures. A mature biofilm could accommodate additional niches and allow for the co-occurrence of a larger variety of metabolisms as the biofilm ages. Furthermore, this type of established biofilm would be able to support bacteria growing under a range of conditions, from aerobic to microaerophilic to anaerobic, and from autotrophic to mixotrophic and heterotrophic (Davey and O’toole, 2000; Stoodley et al., 2002). For example, sequences belonging to the family *Desulfobulbaceae* were not found until day 13 and were only detected above 1% after ∼2.5 months (76E) at Tica and Tamtown (∼5–8 months-old). Cultured representatives from this family are anaerobic, sulfate-reducing bacteria (Savage et al., 2010), which would be expected to thrive at later time points once a more mature biofilm with anaerobic conditions has developed.

In addition, the settlement and immigration of macrofaunal organisms onto the basalt surfaces would further increase microbial diversity by the introduction of macrofaunal-associated microbes and by further increasing possible niches available for microbes. We observed a substantial increase in colonizing macrofauna over time on the experimental panels. For example, at day 4, the experimental panel had five individual macrofaunal colonists; the day 76 experimental panel had 78 colonists, and the 9 months panel (day 293) hosted 122 colonists (Shank, unpublished data). The earliest colonists observed on these panels included foraminifera, and mobile limpets and polychaetes. In contrast, *Tevnia jerichonana* and *Riftia pachyptila* were not detected until 9 months. Interestingly, a gammaproteobacterial tag (Tag.92) sharing 99% sequence identity with the symbiont of *Tevnia jerichonana* (Nelson and Fisher, 2000) was detected prior to observed settlement of *Tevnia jerichonana*. This sequence could have come from the free-living form of the symbiont and may act as a settlement cue for larval *Tevnia jerichonana* recruits. Based on number of sequences detected on these diffuse-flow basalts that are close relatives of known vent symbionts, this environment could be a prime habitat for the free-living form of different symbionts (Harmer et al., 2008; Callac et al., 2013). However, we cannot exclude the possibility that recently settled *Tevnia jerichonana* recruits with incorporated symbionts were present, but not detected upon examination with the dissecting microscope.

We observed evidence of macrofaunal feeding upon well-developed biofilms on early experimental panels in the form of gastropod radula scrapping patterns (days 4 and 13; Supplementary Figure S1E). The grazing activity partially clears the associated biofilms, rejuvenating the biofilm by opening up new surfaces for re-colonization and thus increasing overall diversity, providing a possible mechanism to explain the differences observed among the panels.

#### Microbial Succession: OTU Level Diversity

Although the overall proportions of microbial groupings changed with time, we found the same OTUs at early and late time points, indicating that microbial phylotypes that settle or immigrate onto new surfaces within days of initial exposure can persist for months (>9 months). Our results suggest that early settlers play a key role in determining the composition of the older community. Rather than a new set of OTUs supplanting the initial settlers, it appears that most OTUs establish themselves within days and, while abundances change with time, the overall OTU profile does not. This pattern persists for both the experimental and control locations, and for different classes. For example, 55% of the tag OTUs belonging to the genus *Sulfurovum* were found at both 9E and 293E (17/31) and these OTUs make up >99% of all *Sulfurovum* tags. We hypothesize that the ability to form biofilms would help allow specific phylotypes (OTUs) to persist over time. The biofilm matrix, which is to a large extent composed of exopolymeric substances excreted by the biofilm-forming microbes, like exopolysaccharides (EPSs), offers protection from environmental variations, like pH and temperature, and can also decrease toxicity by binding and sequestering heavy metals contained in the hydrothermal fluid (Edwards et al., 2005; Sievert and Vetriani, 2012). Various *Epsilonproteobacteria* isolates, including *Sulfurovum*, are known to contain genes for biosynthesis of EPSs and for quorum sensing (Sievert and Vetriani, 2012; Pérez-Rodríguez et al., 2014). While *Sulfurovum* taxa were abundant and diverse throughout the experiment, they were especially abundant during the first 2 weeks, comprising 60–80% of each community, making them key players in establishing a persistent biofilm. Interestingly, the most numerically abundant OTU belongs within the genus *Sulfurovum* (Clone.32. Tag.264) and is highly similar (≥98%) to sequences recovered from other active sulfides and diffuse-flow vent sites (Stott et al., 2008; Zhou et al., 2009) and to sequences recovered from inactive sulfides on the EPR (Sylvan et al., 2012b). It is possible that this OTU represents a dominant, widely distributed vent-associated species that is prevalent at active vent sites and that persists at inactive sites by being able to oxidize sulfide minerals contained in the chimneys or, alternatively, its DNA might be preserved and thus could represent a ‘relic’ of past activity.

#### Comparison among Vent Sites and of Pre-eruption with Post-eruption Environments

From our results it is clear that, despite spatial and temporal separation, bacterial communities of similar ages, in similar habitats, are relatively comparable in community composition and distribution. Although separated by ∼0.58 km and sampled 19–28 months apart, bacterial communities from Tica vent and Tamtown [Basalt and 293E or 76E; Trap and 4E] share similar, but not identical, class and genus level phylogeny. The Trap sample is exclusively comprised of *Epsilonproteobacteria* (*Arcobacter, Sulfurovum*, and *Sulfurimonas*), and is similar in composition to day 4E at the experimental pre-eruption site. These samples share >30% of the identified *Epsilonproteobacterial* OTUs, even though they were sampled at different times and locations and represent different substrate types [stainless-steel (Trap) and basalt (day 4E)], suggesting that, at least for early colonization, the material of the provided substrate does not appear to play a major role. The post-eruption Tamtown Basalt as well as the pre-eruption 293E and 76E are comprised of *Sulfurovum, Bacteroidetes, Delta*- and *Gammaproteobacteria*. The volcanic eruption has been dated around the end of 2005/early 2006, with brief pulses of activity still measured through April 2006 (Tolstoy et al., 2006; Soule et al., 2007). This provides a conservative estimate of the age of the Tamtown Basalt community of around 5–8 months, which is comparable in age to 293E. Comparing diversity estimates for these two communities, we find that 293E appears to host a substantially more diverse community than Tamtown Basalt. Most likely, more species immigrated onto the panel samples than the Tamtown Basalt, because they were placed in the midst of an active vent community, with a diverse and thriving macrofaunal community and associated microbes. In contrast, the Tamtown sample represents a natural basalt community obtained from an environment in which the entire community had been destroyed by the volcanic eruption months before. At the time of sampling, there were small tubeworms (*Tevnia jerichonana*; *∼*2 cm in length) colonizing the Tamtown basalt, and *Ctenopelta porifera* limpets in the vicinity, but the macrofaunal community was young and not highly diverse (Nees et al., 2009). In contrast, the 293E experimental panel was colonized with multiple species of polychaetes and gastropods (Shank, unpublished data). The precise timing of when the diffuse-flow venting at Tamtown became active after the eruption is not known. Thus, the Tamtown biological community (native Basalt sample) could potentially be younger than initially estimated if venting did not start immediately after the eruption, thereby explaining why the native Basalt sample diversity estimates are more in line with the earlier panel samples. Still, the entire native Basalt community clusters with that of day 76E and day 293E rather than the earlier panels, suggesting that the site had at least been active for several months.

At the level of specific OTUs, there is extensive overlap, with almost all native Basalt OTUs also detected at the Tica vent site (95% of the OTUs associated with the natural Basalt at Tamtown were also recovered in the experimental panels at Tica; only seven OTUs were exclusive to the native Basalt sample and these OTUs comprise <2% of the total tags for the native Basalt sample). Although these sites are 0.58 km apart, it is possible they are supplied from connected sub-seafloor hydrothermal conduits with similar microbial communities. Additionally, the similar temperature and chemical regimes found in each diffuse-flow habitat may select for identical OTUs. The overlap in community composition, despite the spatial and temporal separation and the fact that the sampling involved both experimental panels and natural basalt communities, is remarkable, and suggests that the microbial communities colonizing new surfaces at diffuse-flow vent sites might follow a predictable successional pattern, paralleling that observed for macrofauna (Shank et al., 1998). Further work is required, however, to confirm this intriguing hypothesis.

Although the Tamtown and Tica communities share many OTUs, they can be distinguished by disparate proportions of two phylogentic groups, *Sulfurovum* and *Bacteroidetes*. The native Basalt hosts a higher proportion of *Sulfurovum* (70% versus 35%), and the lower proportion of *Bacteroidetes* (13% versus 20%) than Tica panel day 293E. While microbial communities at both Tamtown and Tica were likely supported by a combination of chemosynthetic and heterotrophic processes, we propose that the Tamtown native Basalt microbial community was predominantly driven by chemosynthetic processes, while the Tica panel community (day 293E), set in an environment with abundant macrofauna and associated organic matter, was more influenced by heterotrophic processes.

#### Comparison between Biofilm Communities and Diffuse-Flow Fluid Communities: Evidence for Species Sorting

Previous studies have found varying degrees of geographical isolation among bacterial communities in diffuse-flow vent fluids and those associated with plume particles (Opatkiewicz et al., 2009; Huber et al., 2010; Sylvan et al., 2012a), both of which represent the source communities from which the biofilm community is recruited. Opatkiewicz et al. (2009) found considerable overlap in chemical parameters between different vents, yet each vent maintained a consistent and statistically distinct microbial community structure, as determined by TRFLP. One possible explanation for the different findings is that the sites in our study (Tica and Tamtown) had similar chemical parameters and biological indicators (temperatures between 10 and 30°C and presence of similar fauna, i.e., *Tevnia jerichonana*), and were separated by only 0.58 km. Furthermore, Opatkiewicz et al. (2009), Huber et al. (2010), and Sylvan et al. (2012a) all studied the diversity of microbial communities found in diffuse-flow fluids. It is possible that fluid-associated communities reflect the site-specific variations in subsurface diversity, while the selective pressure of establishing a microbial biofilm creates greater community overlap across neighboring sites by means of species sorting. Species sorting occurs when biotic and abiotic environmental conditions select for a subset of bacteria from a larger population, creating a community distinct from the source population (Leibold et al., 2004). Species sorting has been documented as an important biofilm assembly mechanism in fresh water streams (Besemer et al., 2012) and cold seeps (Zhang et al., 2014) among other locations, but has not been studied explicitly at diffuse-flow vent sites.

As further support for species sorting, clear differences between fluid and biofilm associated groups can be identified by comparing our results with published studies of vent fluids. Diffuse-flow fluids from Tica vent hosted abundant and diverse populations of Archaea, as well as gammaproteobacterial *Oceanospirillales* (Campbell et al., 2013), groups which were absent or detected at very low abundances (<<1%) within the biofilms studied here. In our results, the most common gammaproteobacterial sequences on the 9-months panels belong within the *Chromatiales, Methylococcales, Thiotrichales*. Other researchers found *Methylococcales* and *Thiotrichales* tags to be the most common gammaproteobacterial sequences in vent associated mats (Crépeau et al., 2011; Forget and Juniper, 2013), in contrast to vent fluids where the gammaproteobacterial community is dominated by *Pseudoalteromonas* and *Alteromonadales* (Huber et al., 2007).

Surface-associated samples in this study had substantially lower overall diversity than the fluids investigated in other studies, even at Tica vent itself, with Chao-1 values one to two orders of magnitude lower [109–230 (tags:this study); 36,869 (Huber et al., 2007); 2468–4193 (EPR: (Campbell et al., 2013)]. Certainly, higher Chao-1 values can be expected with larger sample size and greater sequencing effort. Yet, intriguingly, even at the same vent location (Tica), when Campbell et al. (2013) winnowed their data to include only vent-specific bacterial, 16S rDNA amplicons they still detected a substantially greater number of observed species (N_obs_:305 this study, maximum N_obs_:168 at 293E) and obtained Chao-1 values higher than our surface-associated estimates (Chao-1:334 95% CI:317–377); this study, Chao-1:230 (95% CI:199–292). Based on our findings, we hypothesize that the process of biofilm formation, on basalt surfaces in diffuse-flow regions, functions as a means for species sorting by selecting for a subset of the fluid-associated microbial community and generating similar surface-associated communities between neighboring vent sites.

## Conclusion

The results from this study contribute to our understanding of the composition and ecology of microbial biofilms in diffuse-flow vent habitats. Not only did we document an increase in microbial diversity over short time scales, the transition from an early biofilm dominated by putative autotrophs to a more diverse late biofilm, but we also identified a pattern of shared OTUs over time and across different vent sites. We propose that the process of biofilm formation selects for a subset of the fluid-associated microbial community and generates similar surface-associated communities. Future studies are needed to address the functional aspects of these communities thereby elucidating the roles of the various phylogenetic groups in biofilm formation and maintenance, and to clarify if the observed micro-diversity, e.g., within the *Sulfurovum* and *Bacteroidetes* group, translates to metabolic and/or niche diversity. In-depth temporal studies will allow us to resolve the patterns of biofilm community development, how spatially separated communities are related, and to investigate the exact role of biofilms as a precursor to macrofaunal settlement and immigration.

## Conflict of Interest Statement

The authors declare that the research was conducted in the absence of any commercial or financial relationships that could be construed as a potential conflict of interest.
