# Supplementary material for: Bacterial diversity and successional patterns during biofilm formation on freshly exposed basalt surfaces at diffuse-flow deep-sea vents
Source: Front Microbiol. 2015 Sep 10;6:901. doi: 10.3389/fmicb.2015.00901 (PMC4564720; doi:10.3389/fmicb.2015.00901)
Supplement: Supplementary file 1 [file Data_Sheet_1.PDF]

Supplementary Material

Bacterial diversity and successional patterns during biofilm formation on freshly exposed basalt surfaces at diffuse-flow deep-sea vents

Lara K. Gulmann[1], Stace E. Beaulieu[1], Timothy M. Shank[1], Kang Ding[2], William E. Seyfried[2], Stefan M. Sievert[1,*]

[1]Biology Department, Woods Hole Oceanographic Institution, Woods Hole, MA 02543

[2]Department of Earth Sciences, University of Minnesota, Minneapolis, Minnesota, 55455, USA

[*]Correspondence:
Dr. Stefan Sievert
Biology Department
Woods Hole Oceanographic Institution
266 Woods Hole Rd
Woods Hole, MA 02543
ssievert@whoi.edu

1.1 Supplementary Tables

Supplementary Table 1A. Taxonomy of Tag OTUs. *Epsilonproteobacteria*

#OTU ID	Day9E	Day13E	Day76E	Day293E	Basalt	Day9C	Day13C	Day283C	Taxa	Detected in clone library
Tag.342	798	57	7	9	18	248	235	0	*Epsilonproteobacteria; Campylobacterales; Arcobacter*	yes
Tag.151	0	62	15	49	1	0	0	0		
Tag.54	0	0	0	1	0	0	0	0		
Tag.19	1	2	1	2	0	6	1	0		
Tag.291	0	0	2	1	1	5	23	0		
Tag.3	4	1	0	0	0	2	0	0		
Tag.125	17	2	1	0	0	16	44	0		
Tag.16	30	74	267	279	81	7	0	6	*Epsilonproteobacteria;Campylobacterales;Campylobacteraceae;Sulfurospirillum*	yes
Tag.197	4	39	174	40	0	0	0	0		
Tag.119	1	5	1	2	1	5	4	4		
Tag.10	61	6	1	5	1	191	50	4	*Epsilonproteobacteria;Campylobacterales;Helicobacteraceae;Sulfurimonas*	yes
Tag.59	1	0	0	0	0	27	2	2		
Tag.140	1	0	0	0	2	63	17	1		
Tag.190	4	1	0	1	4	0	0	0		
Tag.229	6	7	2	4	61	34	4	0		
Tag.261	86	10	5	1	0	159	35	1		
Tag.339	4	23	5	9	1	0	2	0		
Tag.13	0	0	0	0	0	15	7	0		
Tag.46	0	1	0	1	0	4	0	0		
Tag.94	0	2	0	2	0	0	0	0		
Tag.175	0	0	0	0	0	23	0	0		
Tag.321	0	0	0	0	0	0	0	1		
Tag.328	2	0	0	0	0	1	0	0		
Tag.90	0	0	0	0	0	2	3	0	*Epsilonproteobacteria;Campylobacterales;Helicobacteraceae;Sulfurovum*	yes
Tag.107	0	3	2	1	1	0	0	0		
Tag.264	965	495	315	353	57	826	585	50		
Tag.11	216	285	88	217	718	82	85	44		
Tag.178	11	710	450	156	18	6	2	1		
Tag.346	43	176	25	41	8	18	24	3		
Tag.230	225	286	4	33	1996	33	21	5		
Tag.344	3	134	41	24	76	10	6	6		
Tag.277	1	7	1	20	0	3	0	1		
Tag.17	1	107	68	13	33	1	0	0		
Tag.196	392	72	4	11	281	617	599	24		
Tag.227	7	7	4	11	3	8	1	1		
Tag.49	3	3	0	9	0	4	0	0		
Tag.270	6	2	2	3	0	22	6	5		
Tag.102	1	4	1	2	0	0	0	0		
Tag.204	2	2	0	2	50	0	0	0		
Tag.4	55	9	0	1	19	161	50	3		
Tag.73	0	6	0	1	0	0	0	0		
Tag.111	2	5	0	1	1	1	0	0		
Tag.117	0	11	0	1	21	3	4	0		
Tag.239	3	6	0	1	6	6	1	2		
Tag.254	0	1	0	1	10	0	0	0		
Tag.60	0	0	0	0	0	1	0	1		
Tag.67	5	0	1	0	1	0	0	0		
Tag.76	1	1	0	0	5	0	0	1		
Tag.100	1	0	0	0	0	2	0	0		
Tag.153	1	0	0	0	0	5	0	0		
Tag.214	0	0	0	0	4	0	0	0		
Tag.233	1	0	0	0	0	1	1	0		
Tag.256	0	0	0	0	0	3	1	1		
Tag.282	0	0	0	0	4	0	0	0		
Tag.121	0	4	7	10	2	0	0	0	*Epsilonproteobacteria;Campylobacterales*	yes
Tag.126	5	2	2	5	2	2	0	1		
Tag.161	3	3	2	5	1	1	2	1		
Tag.88	0	0	1	3	0	2	0	0		
Tag.0	0	0	8	2	0	1	0	0		
Tag.36	2	14	1	1	4	0	0	0		
Tag.158	3	9	0	1	0	0	0	1		
Tag.50	0	1	0	0	0	0	0	0		
Tag.37	0	0	0	0	0	1	0	0		
Tag.323	1	0	1	0	0	9	1	0		

Supplementary Table 1B. Taxonomy of Tag OTUs. *Gammaproteobacteria*

#OTU ID	Day9E	Day13E	Day76E	Day293E	Basalt	Day9C	Day13C	Day283C	Taxa	Detected in clone library
Tag.92	0	0	1	221	29	4	1	2	*Gammaproteobacteria;Chromatiales*	yes
Tag.276	0	0	0	0	0	6	0	179	*Gammaproteobacteria; Methylococcales;Methylococcaceae*	yes
Tag.232	0	0	0	0	0	0	0	1		
Tag.91	0	0	0	0	0	1	0	0		
Tag.258	0	0	0	2	0	0	0	8		
Tag.24	0	0	1	51	0	5	0	450	*Gammaproteobacteria; Thiotrichales*	yes
Tag.172	1	0	4	30	0	5	1	14		
Tag.43	0	0	0	4	0	0	0	0		
Tag.187	0	0	0	0	0	3	4	0		
Tag.265	3	0	9	6	1	4	0	142	*Gammaproteobacteria;Thiotrichales;Thiotrichaceae;Leucothrix*	yes
Tag.42	0	0	0	0	8	0	0	1		
Tag.177	0	0	12	5	0	1	0	18		
Tag.274	0	2	1	5	0	0	0	6		
Tag.186	0	0	0	1	0	3	2	0		
Tag.243	0	0	3	74	0	5	2	481	*Gammaproteobacteria; Thiotrichales;Thiotrichaceae*	yes
Tag.83	0	0	0	0	0	0	0	74		
Tag.81	0	0	0	0	0	22	1	6		
Tag.353	0	0	0	0	0	0	0	1		
Tag.210	0	0	0	0	0	2	0	8	*Gammaproteobacteria; Thiotrichales; Piscirickettsiaceae*	no
Tag.262	1	0	0	1	0	5	0	8		
Tag.279	2	0	2	0	0	0	1	0	*Gammaproteobacteria;Enterobacteriales; Enterobacteriaceae*	no
Tag.101	21	0	0	0	0	49	51	6	*Gammaproteobacteria; Alteromonadales; Colwelliaceae; Colwellia*	no
Tag.247	0	2	0	0	0	1	3	0		
Tag.182	0	0	0	0	0	19	7	4		
Tag.2	0	0	0	0	0	2	0	7	*Gammaproteobacteria; Alteromonadales*	no
Tag.235	0	0	0	0	0	0	0	2		
Tag.307	0	0	0	0	0	1	0	1	*Gammaproteobacteria; Alteromonadales; Pseudoalteromonadaceae*	no
Tag.260	0	0	0	0	0	1	0	0	*Gammaproteobacteria; Alteromonadales; Psychromonadaceae; Psychromonas*	no
Tag.221	0	0	0	0	0	1	0	0	*Gammaproteobacteria; Legionellales*	no
Tag.55	0	0	0	0	0	3	7	0	*Gammaproteobacteria; Oceanospirillales; Oceanospirillaceae*	no
Tag.335	0	0	0	0	0	11	0	1	*Gammaproteobacteria; Oceanospirillales; SUP05*	no
Tag.246	0	0	0	0	0	1	0	0	*Gammaproteobacteria; Pseudomonadales; Moraxellaceae; Acinetobacter*	no
Tag.341	0	0	0	0	0	2	0	0		
Tag.25	0	1	0	1	0	3	7	2	*Gammaproteobacteria; Pseudomonadales; Pseudomonadaceae*	no
Tag.199	0	0	0	0	0	0	0	13	*Gammaproteobacteria*	yes
Tag.207	0	0	0	0	0	1	1	3		
Tag.18	0	0	0	0	0	0	0	2		
Tag.62	0	0	0	0	0	0	0	1		
Tag.136	0	0	0	0	0	1	0	1		
Tag.286	0	0	1	0	0	0	0	1		
Tag.292	0	0	0	0	0	0	0	1		
Tag.294	0	0	0	0	0	0	0	1		
Tag.295	0	0	0	0	0	0	0	1		
Tag.321	0	0	0	0	0	0	0	1		
Tag.343	0	0	0	0	0	0	0	1		
Tag.297	0	0	0	0	0	1	0	0		
Tag.316	0	0	0	0	0	1	0	0		
Tag.95	0	0	0	0	0	0	0	1		

Supplementary Table 1C. Taxonomy of Tag OTUs. *Bacteroidetes*

#OTU ID	Day9E	Day13E	Day76E	Day293E	Basalt	Day9C	Day13C	Day283C	Taxa	Detected in clone library
Tag.6	2	19	25	27	33	5	1	0	*Bacteroidetes*	yes
Tag.20	0	0	0	1	0	0	0	0		
Tag.77	0	0	0	0	1	0	0	0		
Tag.82	0	0	0	0	3	0	0	0		
Tag.93	0	0	0	0	0	0	0	1		
Tag.96	0	0	0	0	1	0	0	0		
Tag.99	0	1	0	5	0	0	1	0		
Tag.120	0	1	0	6	0	0	0	0		
Tag.164	0	0	1	1	2	0	0	0		
Tag.167	0	0	0	0	0	1	0	0		
Tag.192	0	0	0	2	0	1	0	28		
Tag.195	0	0	0	0	0	1	0	0		
Tag.218	0	0	0	0	0	2	0	4		
Tag.225	0	0	1	4	2	1	0	0		
Tag.236	0	0	4	6	6	0	0	0		
Tag.244	0	6	14	6	21	0	0	1		
Tag.255	0	0	0	0	1	0	0	0		
Tag.257	1	9	0	2	4	1	0	0		
Tag.310	0	0	0	0	6	0	0	0		
Tag.317	1	3	3	3	4	2	0	2		
Tag.332	0	1	0	1	7	0	0	0		
Tag.370	0	0	1	0	0	0	0	0		
Tag.32	0	2	0	0	0	0	0	0	*Bacteroidetes; Bacteroidia*	no
Tag.242	0	31	85	26	50	0	0	0		
Tag.78	7	3	4	7	14	2	0	2	*Bacteroidetes; Bacteroidia; Bacteroidales*	yes
Tag.157	0	1	1	1	10	0	0	0		
Tag.159	0	6	6	10	1	0	0	0		
Tag.216	0	1	0	2	0	0	0	0		
Tag.372	0	1	0	0	0	0	0	0		
Tag.33	0	1	0	3	1	1	0	1		
Tag.40	0	0	0	0	1	0	0	0		
Tag.45	0	1	5	35	49	2	0	0		
Tag.128	0	0	2	3	0	0	0	0		
Tag.147	0	0	0	2	0	0	0	0		
Tag.189	0	0	0	0	3	0	0	0		
Tag.203	0	0	0	6	0	0	0	0		
Tag.219	0	0	0	3	0	0	0	0		
Tag.269	0	2	0	0	0	0	0	0		
Tag.303	0	0	0	5	0	1	0	0		
Tag.324	0	0	3	8	2	0	0	1		
Tag.337	0	0	0	0	8	0	0	0		
Tag.28	1	0	4	12	1	2	0	0	*Bacteroidetes; Bacteroidia; Bacteroidales;Marinilabiaceae*	yes
Tag.39	0	1	6	21	0	1	0	0		
Tag.314	0	2	13	84	1	5	1	0		
Tag.165	0	0	4	6	31	0	0	0	*Bacteroidetes; Bacteroidia; Bacteroidales;Marinilabiaceae:VC21_Bac22*	yes
Tag.220	0	0	0	3	2	0	0	0		
Tag.253	0	1	1	11	1	1	0	1		
Tag.300	0	0	2	15	1	1	0	0		
Tag.160	0	0	0	0	0	0	0	1	*Bacteroidetes; Flavobacteriia; Flavobacteriales:Flavobacteriales*	yes
Tag.217	1	0	0	0	0	2	1	0		
Tag.245	0	0	0	0	0	2	0	0		
Tag.305	0	1	0	0	4	0	0	0		
Tag.360	0	0	0	0	0	0	0	33		
Tag.133	0	0	0	0	0	0	0	1	*Bacteroidetes; Flavobacteriia; Flavobacteriales*	yes
Tag.155	9	0	0	0	0	4	5	0		
Tag.213	4	0	0	1	0	9	0	1		
Tag.215	23	9	6	23	4	6	5	1		
Tag.228	0	0	0	0	0	10	6	6		
Tag.281	0	0	0	0	0	1	0	1		
Tag.329	0	0	0	0	0	4	0	0		
Tag.5	0	0	0	0	0	0	0	1	*Bacteroidetes; Flavobacteriia; Flavobacteriales:Flavobacteriales:Flavobacteriaceae*	yes
Tag.12	0	0	0	1	0	0	0	0		
Tag.70	0	0	0	0	0	1	0	0		
Tag.169	6	25	86	32	117	10	0	67		
Tag.205	0	8	20	8	7	0	0	0		
Tag.224	0	0	0	0	0	0	0	3		
Tag.298	1	0	0	0	0	3	1	7		
Tag.315	0	0	0	0	1	0	0	0		
Tag.322	5	4	1	0	13	0	1	0		
Tag.123	0	0	0	0	0	10	0	12		
Tag.179	0	3	10	17	0	0	0	0		
Tag.222	3	0	0	0	0	1	0	0		
Tag.267	0	2	3	21	94	0	0	0		
Tag.318	3	1	7	7	63	1	0	212		
Tag.338	0	0	0	0	0	0	0	4		
Tag.34	0	0	0	1	0	0	0	0	*Bacteroidetes; Sphingobacteriia; Sphingobacteriales; Ekhidnaceae*	yes
Tag.51	0	0	0	2	0	0	1	0		
Tag.146	0	0	1	4	0	1	1	0		
Tag.359	0	0	0	0	0	1	0	0		
Tag.105	0	0	0	0	0	1	0	111	*Bacteroidetes; Sphingobacteriia; Sphingobacteriales;Saprospiraceae*	yes
Tag.113	0	0	0	0	0	0	0	7		
Tag.163	0	1	0	0	0	0	0	4		
Tag.168	0	0	0	0	0	0	0	1		
Tag.181	0	0	1	3	0	0	0	3		
Tag.184	0	0	1	3	0	0	0	10		
Tag.202	0	0	0	1	0	0	0	2		
Tag.293	0	0	0	8	0	0	0	2		
Tag.309	0	0	0	1	39	0	0	1		
Tag.347	0	0	0	0	0	0	0	13		
Tag.357	0	0	4	12	0	0	0	1		
Tag.361	0	0	0	0	0	1	0	2		
Tag.80	0	0	0	0	0	4	1	0	*Bacteroidetes; Sphingobacteriia; Sphingobacteriales;Saprospiraceae;Aureispira*	no
Tag.191	0	0	0	0	0	0	3	0		
Tag.44	0	0	0	0	0	1	0	0	*Bacteroidetes; Sphingobacteriia; Sphingobacteriales;Saprospiraceae;Lewinella*	no
Tag.251	0	0	0	0	0	1	0	1		
Tag.108	0	0	0	0	0	2	0	2	*Bacteroidetes; Flavobacteriia; Flavobacteriales:Cryomorphaceae*	no
Tag.231	7	0	0	0	0	10	3	0		
Tag.238	34	1	0	1	0	28	31	7		
Tag.289	0	0	0	0	0	2	1	5	*Bacteroidetes; Flavobacteriia; Flavobacteriales:Flavobacteriales:Flavobacteriaceae:Kordia*	no
Tag.241	0	0	5	16	0	0	0	35	*Bacteroidetes; Flavobacteriia; Flavobacteriales:Flavobacteriales:Flavobacteriaceae:Lutimonas*	no
Tag.249	5	0	6	11	0	6	0	61		
Tag.271	0	1	0	7	0	2	1	4		
Tag.352	0	0	0	0	1	0	0	0		
Tag.362	0	0	0	0	0	0	0	15	*Bacteroidetes; Flavobacteriia; Flavobacteriales:Flavobacteriales:Flavobacteriaceae:Olleya*	no
Tag.252	0	0	0	0	0	4	3	3	*Bacteroidetes; Flavobacteriia; Flavobacteriales:Flavobacteriales:Flavobacteriaceae:Tenacibaculum*	no
Tag.268	0	0	1	0	1	2	0	2		
Tag.355	0	0	0	1	0	0	0	0	*Bacteroidetes; Sphingobacteriia; Sphingobacteriales*	
Tag.127	0	0	0	0	0	1	0	0	*Bacteroidetes; Sphingobacteriia; Sphingobacteriales; Ekhidnaceae:JTB248*	no

Supplementary Table 1D. Taxonomy of Tag OTUs. *Deltaproteobacteria*

#OTU ID	Day9E	Day13E	Day76E	Day293E	Basalt	Day9C	Day13C	Day283C	Taxa	Detected in clone library
Tag.30	0	0	3	2	1	0	0	0	*Deltaproteobacteria*	yes
Tag.124	0	0	0	0	1	0	0	0		
Tag.139	0	0	6	0	0	0	0	0		
Tag.176	2	1	21	19	13	2	0	2		
Tag.266	0	0	0	0	0	0	0	1		
Tag.330	0	3	7	3	9	1	0	0	*Deltaproteobacteria; Desulfobacterales; Desulfobulbaceae*	yes
Tag.311	0	0	1	5	0	0	0	1		
Tag.327	0	0	1	5	0	0	0	0		
Tag.364	0	0	5	6	0	0	0	1		
Tag.134	0	3	161	34	150	0	0	2	*Deltaproteobacteria; Desulfobacterales; Desulfobulbaceae;Desulfocapsa*	yes
Tag.272	0	0	41	7	210	0	0	0		
Tag.302	0	1	0	0	0	0	0	0	*Deltaproteobacteria;Desulfuromonadales*	yes
Tag.154	0	2	25	24	12	1	0	1	*Deltaproteobacteria;Desulfuromonadales;Desulfuromonadaceae*	yes
Tag.129	0	0	0	0	0	0	0	1	*Deltaproteobacteria;GMD14H09*	yes
Tag.148	0	0	1	0	0	0	0	0		
Tag.365	0	1	0	0	16	0	0	0		
Tag.56	0	0	1	0	0	0	0	0	*Deltaproteobacteria; Myxococcales*	yes
Tag.137	0	0	1	0	0	0	0	0		
Tag.180	0	0	0	0	0	0	0	1		
Tag.319	0	0	0	0	0	0	0	1		
Tag.35	0	0	0	1	0	0	0	0	*Deltaproteobacteria; Syntrophobacterales; Desulfobacteraceae*	yes
Tag.185	0	0	0	3	7	0	0	0		
Tag.306	0	0	0	3	0	0	0	0		
Tag.226	0	0	0	4	2	0	0	0		
Tag.308	1	3	0	2	2	1	0	2	*Deltaproteobacteria;Bdellovibrionales; Bacteriovoracaceae*	no
Tag.237	0	0	0	0	0	7	2	0	*Deltaproteobacteria;Bdellovibrionales; Bacteriovoracaceae;Bacteriovorax*	no
Tag.162	0	0	0	0	0	0	0	1	*Deltaproteobacteria;Bdellovibrionales; Bacteriovoracaceae;Bdellovibrio*	no
Tag.371	0	2	13	11	4	2	0	0	*Deltaproteobacteria; Desulfobacterales; Desulfobulbaceae;Desulfobulbus*	no
Tag.208	0	1	34	0	4	0	0	0	*Deltaproteobacteria;Desulfuromonadales;Desulfuromonas*	no
Tag.122	0	0	0	1	0	0	0	6	*Deltaproteobacteria; Myxococcales; Haliangiaceae*	no
Tag.8	0	0	0	0	0	0	0	2	*Deltaproteobacteria;Myxococcales;OM27*	no
Tag.85	0	0	0	0	1	0	0	0		
Tag.250	0	0	1	0	0	0	0	0		
Tag.287	0	0	0	0	0	0	1	0		
Tag.368	0	0	0	0	0	0	0	2		
Tag.275	0	0	0	0	1	0	0	3	*Deltaproteobacteria; NB1-j;JTB38*	no
Tag.288	0	0	0	0	0	1	0	0		
Tag.171	0	0	4	1	5	0	0	0	*Deltaproteobacteria; PB19*	no
Tag.259	0	0	0	0	0	0	0	7		
Tag.278	0	0	0	2	0	0	0	0		
Tag.373	0	0	0	0	0	1	0	0	*Deltaproteobacteria; Spirobacillales*	no
Tag.112	0	0	0	0	1	0	0	0	*Deltaproteobacteria; Syntrophobacterales; Desulfobacteraceae; Desulfobacterium*	no
Tag.22	0	0	0	0	1	0	0	0	*Deltaproteobacteria; Syntrophobacterales; Desulfobacteraceae; Desulfococcus*	no

Supplementary Table 2A. Taxonomy of Clone OTUs. *Epsilonproteobacteria*

#OTU ID	Trap	Day4E	Day9E	Day13E	Day76E	Day293E	Basalt	Day9C	Day13C	Day283C	Taxa
Clone.14	1	1	0	0	0	0	0	0	0	0	*Epsilonproteobacteria; Campylobacterales; Arcobacter*
Clone.22	0	0	1	0	0	0	0	0	0	0	
Clone.75	1	0	0	0	0	0	0	0	2	0	
Clone.96	1	0	0	0	0	0	0	0	0	0	
Clone.101	27	11	9	0	0	1	0	0	8	0	
Clone.104	0	1	0	0	0	0	0	0	0	0	
Clone.122	0	1	0	3	1	0	0	0	0	0	
Clone.11	0	0	0	0	1	1	0	0	0	0	*Epsilonproteobacteria;Campylobacterales;Campylobacteraceae;Sulfurospirillum*
Clone.47	0	0	0	0	3	3	0	0	0	0	
Clone.87	0	0	0	0	1	0	0	0	0	0	
Clone.99	0	0	0	2	0	1	0	0	1	0	
Clone.131	0	0	0	0	5	3	0	0	0	0	
Clone.74	0	2	0	0	0	0	0	0	0	0	*Epsilonproteobacteria;Campylobacterales;Helicobacteraceae;Sulfurimonas*
Clone.88	0	0	0	0	0	0	0	0	1	0	
Clone.20	0	3	0	0	0	0	0	0	0	0	
Clone.24	1	0	0	0	0	0	1	0	0	0	
Clone.27	0	0	0	0	0	2	0	0	0	0	
Clone.43	2	4	7	0	0	0	0	1	6	0	
Clone.54	4	1	0	0	0	0	0	0	0	0	
Clone.76	1	0	0	0	0	0	0	0	0	0	
Clone.80	0	2	0	1	0	0	0	0	0	0	
Clone.81	0	0	0	0	0	0	1	0	0	0	
Clone.106	0	0	1	0	0	0	0	0	0	0	
Clone.109	0	0	0	0	0	0	1	0	0	0	
Clone.128	0	3	0	0	0	0	0	0	0	0	
Clone.2	0	0	0	0	0	1	0	0	0	0	*Epsilonproteobacteria;Campylobacterales;Helicobacteraceae;Sulfurovum*
Clone.5	0	0	0	5	3	0	0	0	0	0	
Clone.8	0	0	0	4	3	0	1	0	0	0	
Clone.9	0	0	0	1	0	0	1	0	0	0	
Clone.15	0	0	0	0	0	0	0	0	1	0	
Clone.17	0	0	3	0	0	0	1	0	0	0	
Clone.21	0	0	0	1	0	0	0	0	0	0	
Clone.29	0	0	0	1	0	0	0	0	0	0	
Clone.30	1	0	0	0	0	0	0	0	0	0	
Clone.32	15	4	17	10	6	2	1	34	14	0	
Clone.37	0	0	11	0	3	0	2	0	3	0	
Clone.42	0	1	0	0	0	0	0	0	0	0	
Clone.50	0	0	0	0	0	1	0	0	0	0	
Clone.53	0	0	0	0	0	0	0	2	2	0	
Clone.57	0	0	0	0	0	0	0	1	0	0	
Clone.60	0	0	0	1	0	0	0	0	0	0	
Clone.61	0	0	0	1	0	0	1	2	4	0	
Clone.62	0	1	0	4	4	1	0	0	0	0	
Clone.63	1	0	0	0	0	0	0	0	0	0	
Clone.73	0	0	1	1	0	2	0	0	0	0	
Clone.77	0	0	0	0	0	0	0	1	0	0	
Clone.83	0	0	0	0	0	0	0	0	1	0	
Clone.85	0	11	0	0	0	0	0	4	1	0	
Clone.95	0	0	0	3	0	0	13	0	0	0	
Clone.98	4	0	0	0	0	0	0	0	0	0	
Clone.108	4	0	0	0	0	0	0	0	0	0	
Clone.112	0	0	1	0	0	0	0	0	0	0	
Clone.113	0	2	2	9	14	0	1	0	0	0	
Clone.118	0	0	0	0	0	0	0	1	0	0	
Clone.119	0	0	0	0	2	0	0	0	0	0	
Clone.120	0	2	0	0	0	0	0	0	0	0	

Supplementary Table 2B. Taxonomy of Clone OTUs. *Gammaproteobacteria*

#OTU ID	Trap	Day4E	Day9E	Day13E	Day76E	Day293E	Basalt	Day9C	Day13C	Day283C	Taxa
Clone.72	0	0	0	0	0	3	0	0	0	0	*Gammaproteobacteria;Chromatiales*
Clone.86	0	0	0	0	0	0	0	0	0	1	
Clone.4	0	0	0	0	0	0	0	0	0	4	*Gammaproteobacteria; Methylococcales;Methylococcaceae*
Clone.33	0	0	0	0	0	0	0	0	0	1	
Clone.58	0	0	0	0	0	0	0	0	0	1	*Gammaproteobacteria; Thiotrichales*
Clone.48	0	0	0	0	0	2	0	0	0	0	
Clone.1	0	0	0	0	0	2	0	0	0	18	*Gammaproteobacteria; Thiotrichales;Thiotrichaceae*
Clone.66	0	0	0	0	0	0	0	0	0	10	
Clone.40	0	0	0	0	0	0	0	0	0	2	*Gammaproteobacteria; Thiotrichales; Thiotrichaceae; Leucothrix*
Clone.46	0	0	0	0	0	0	0	0	0	12	
Clone.56	0	0	0	0	1	2	0	0	0	0	
Clone.89	0	1	0	0	1	0	0	1	0	2	
Clone.94	0	0	0	0	0	0	13	0	0	0	

Supplementary Table 2C. Taxonomy of Clone OTUs. *Bacteroidetes*

#OTU ID	Trap	Day4E	Day9E	Day13E	Day76E	Day293E	Basalt	Day9C	Day13C	Day283C	Taxa
Clone.79	0	0	0	0	0	0	1	0	0	0	*Bacteroidetes*
Clone.23	0	0	0	0	0	0	1	0	0	0	*Bacteroidetes; Bacteroidia; Bacteroidales*
Clone.25	0	0	0	0	0	1	0	0	0	0	
Clone.52	0	0	0	0	0	1	0	0	0	0	
Clone.71	0	0	0	0	0	1	0	0	0	0	
Clone.100	0	0	0	0	0	0	1	0	0	0	
Clone.107	0	0	0	0	0	0	2	0	0	0	
Clone.111	0	0	0	0	0	1	1	0	0	0	
Clone.69	0	1	0	0	0	1	0	0	0	0	*Bacteroidetes; Bacteroidia; Bacteroidales;Marinilabiaceae*
Clone.93	0	0	0	0	0	2	0	0	0	0	
Clone.70	0	0	0	0	0	3	0	0	0	0	*Bacteroidetes; Bacteroidia; Bacteroidales;Marinilabiaceae:VC21_Bac22*
Clone.82	0	0	0	0	0	0	1	0	0	0	
Clone.44	0	1	0	0	0	0	0	0	0	0	*Bacteroidetes; Flavobacteriia; Flavobacteriales*
Clone.116	0	0	0	2	0	0	0	0	0	0	
Clone.6	0	0	0	0	0	0	0	0	0	1	*Bacteroidetes; Flavobacteriia; Flavobacteriales:Flavobacteriales:Flavobacteriaceae*
Clone.16	0	0	0	0	0	0	3	0	0	0	
Clone.39	0	0	0	0	0	0	1	0	0	0	
Clone.90	0	0	0	0	1	0	0	0	0	0	
Clone.102	0	0	0	0	0	0	1	0	0	0	
Clone.105	0	0	0	0	0	0	5	0	0	0	
Clone.124	0	0	0	0	1	0	0	0	0	0	
Clone.13	0	0	0	0	0	1	0	0	0	0	*Bacteroidetes; Sphingobacteriia; Sphingobacteriales; Ekhidnaceae*
Clone.28	0	0	0	0	0	1	0	0	0	0	*Bacteroidetes; Sphingobacteriia; Sphingobacteriales;Saprospiraceae*
Clone.125	0	0	0	0	0	0	0	0	0	1	

Supplementary Table 2D. Taxonomy of Clone OTUs. *Deltaproteobacteria*

#OTU ID	Trap	Day4E	Day9E	Day13E	Day76E	Day293E	Basalt	Day9C	Day13C	Day283C	Taxa
Clone.12	0	0	0	0	0	1	0	0	0	0	*Deltaproteobacteria*
Clone.10	0	0	0	0	0	1	0	0	0	1	*Deltaproteobacteria; Desulfobacterales; Desulfobulbaceae*
Clone.68	0	0	0	0	0	1	0	0	0	0	
Clone.123	0	0	0	0	0	1	0	0	0	0	
Clone.126	0	0	0	0	0	1	0	0	0	0	
Clone.3	0	0	0	0	0	0	1	0	0	0	*Deltaproteobacteria; Desulfobacterales; Desulfobulbaceae;Desulfocapsa*
Clone.45	0	0	0	0	1	0	4	0	0	0	
Clone.132	0	0	0	0	0	0	2	0	0	0	
Clone.49	0	0	0	0	1	0	0	0	0	0	*Deltaproteobacteria;Desulfurellales*
Clone.65	0	0	0	0	0	1	0	0	0	0	*Deltaproteobacteria;Desulfuromonadales;Desulfuromonadaceae*
Clone.110	0	0	0	0	1	0	0	0	0	0	*Deltaproteobacteria;GMD14H09*
Clone.127	0	0	0	0	0	1	0	0	0	0	*Deltaproteobacteria; Myxococcales*
Clone.41	0	0	0	0	2	0	1	0	0	0	*Deltaproteobacteria; Syntrophobacterales; Desulfobacteraceae*
Clone.67	0	0	0	0	0	1	0	0	0	0	

2.2. Supplementary Figures

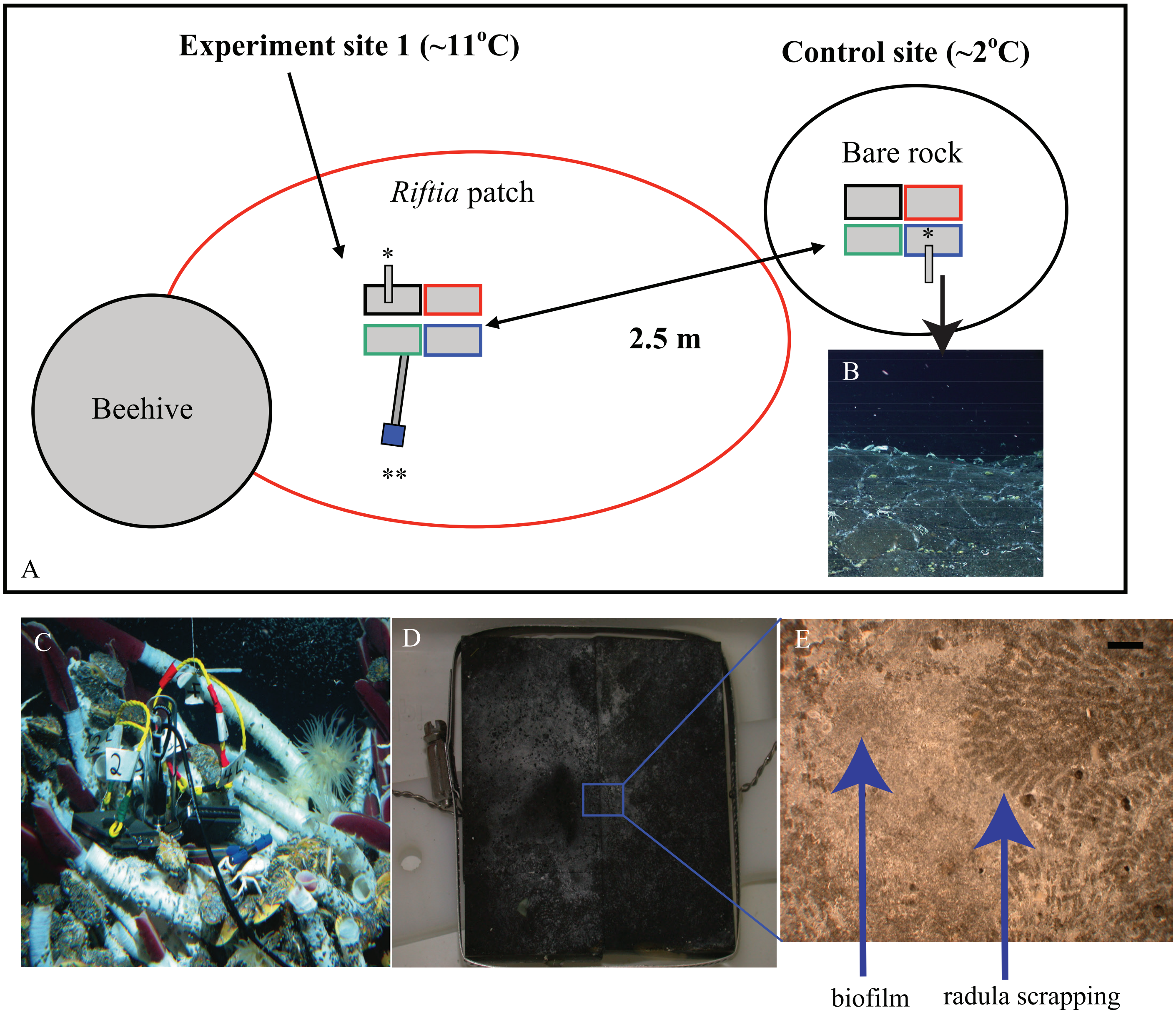

Supplementary Figure 1. (A) Schematic of basalt panels. [* - Time series temperature sensors (VEMCO), ** - *in situ* chemical sensor]. (B) Photograph of Control site at time of deployment. (C) Photograph of Experimental site at time of deployment. (D) Day 4E basalt block with visible biofilm. (E) Dissecting microscope image (8X) of Day 4E basalt block with visible biofilm and probable radula scrapping pattern from hydrothermal vent gastropods. Scale bar is 1.0 mm.

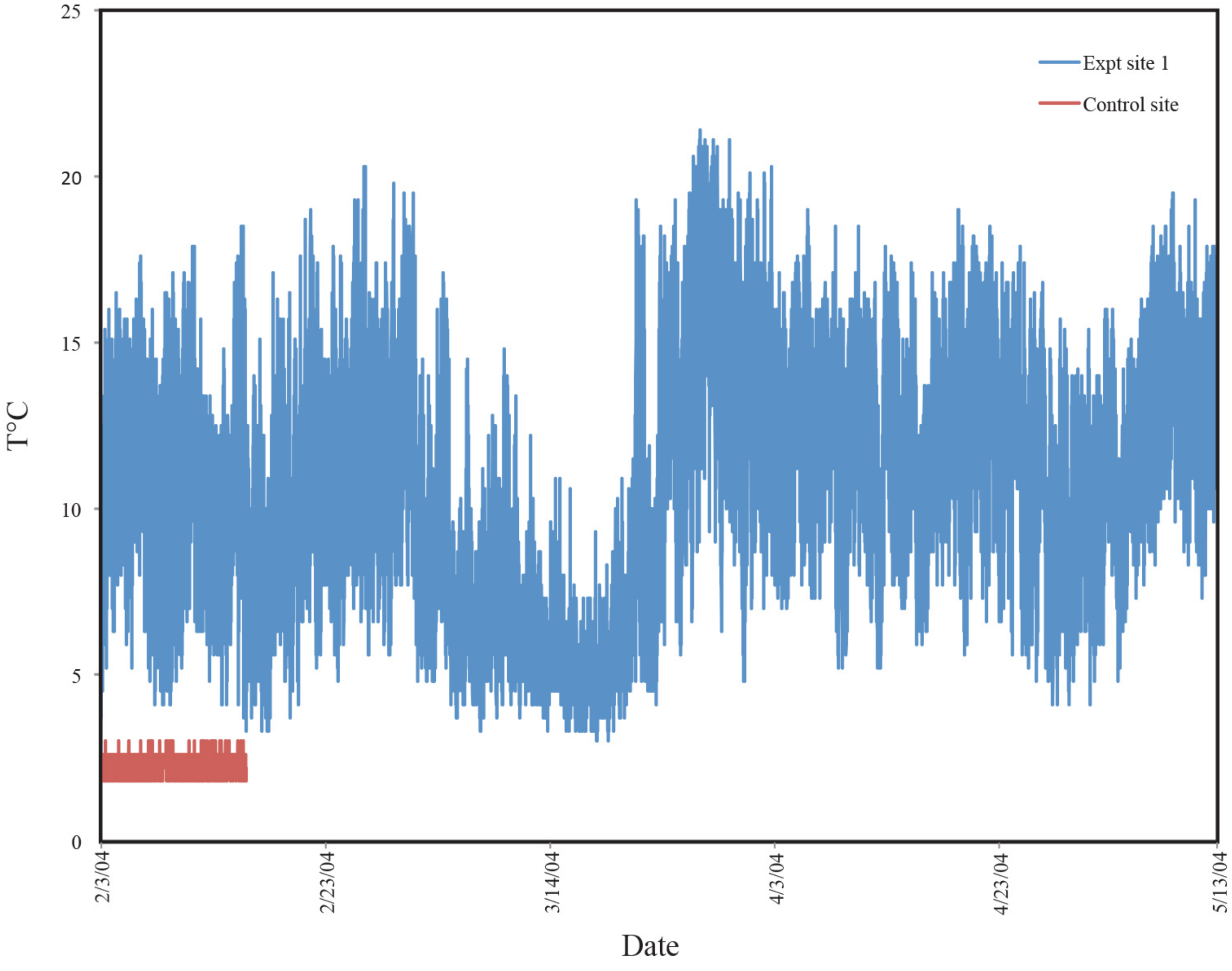

Supplementary Figure 2. VEMCO temperature for Experiment site 1 and Control site.

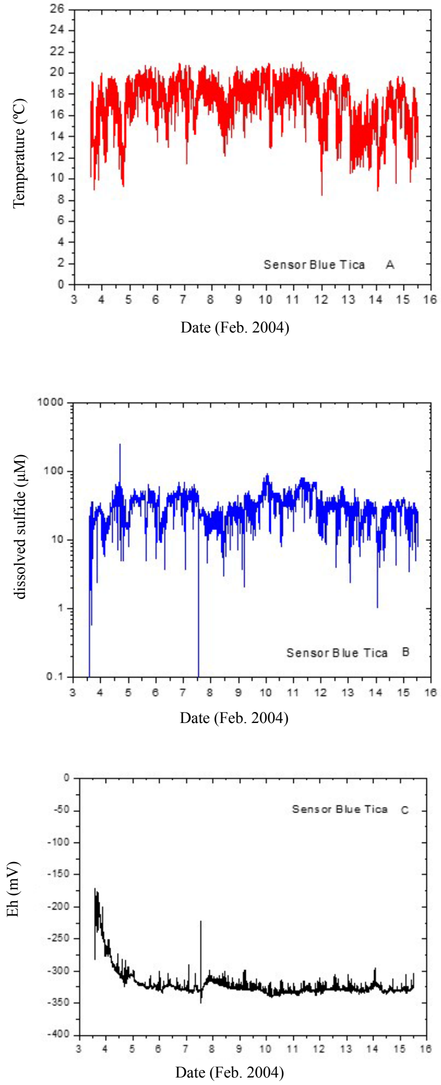

Supplementary Figure 3. Data from *in situ* chemical sensor at Experiment site 1 (A) Temperature (B) dissolved sulfide (C) Eh.

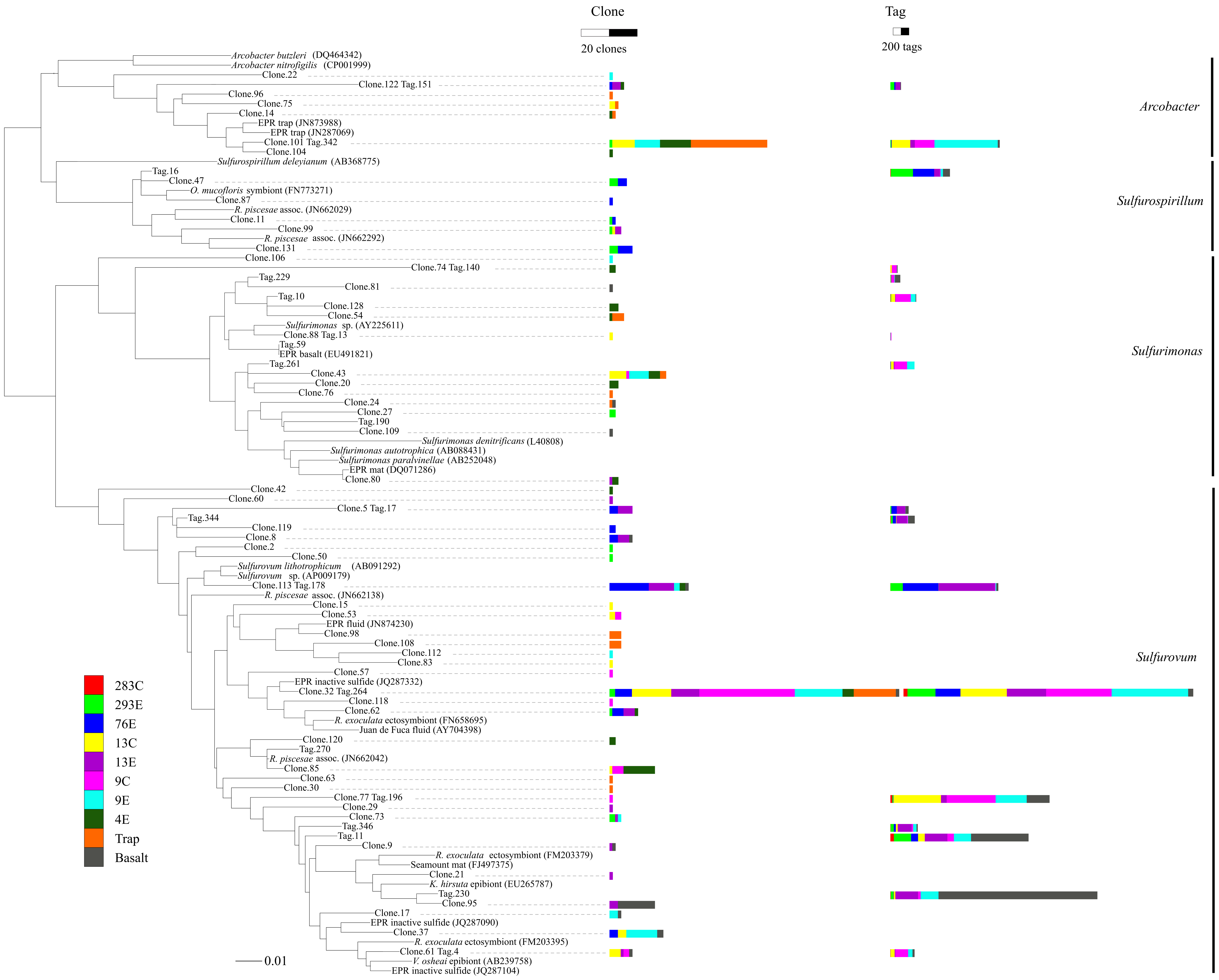

Supplementary Figure 4A. *Epsilonproteobacteria* phylogenetic tree. Trees were constructed using the neighbor-joining algorithm from aligned, full-length clone sequences using the ARB software package. Tag sequences found at high abundances (>50 tags detected per sample), as well as those that were identical to a clone sequence or those that represent a unique lineage were inserted into trees the using parsimony insertion tool.

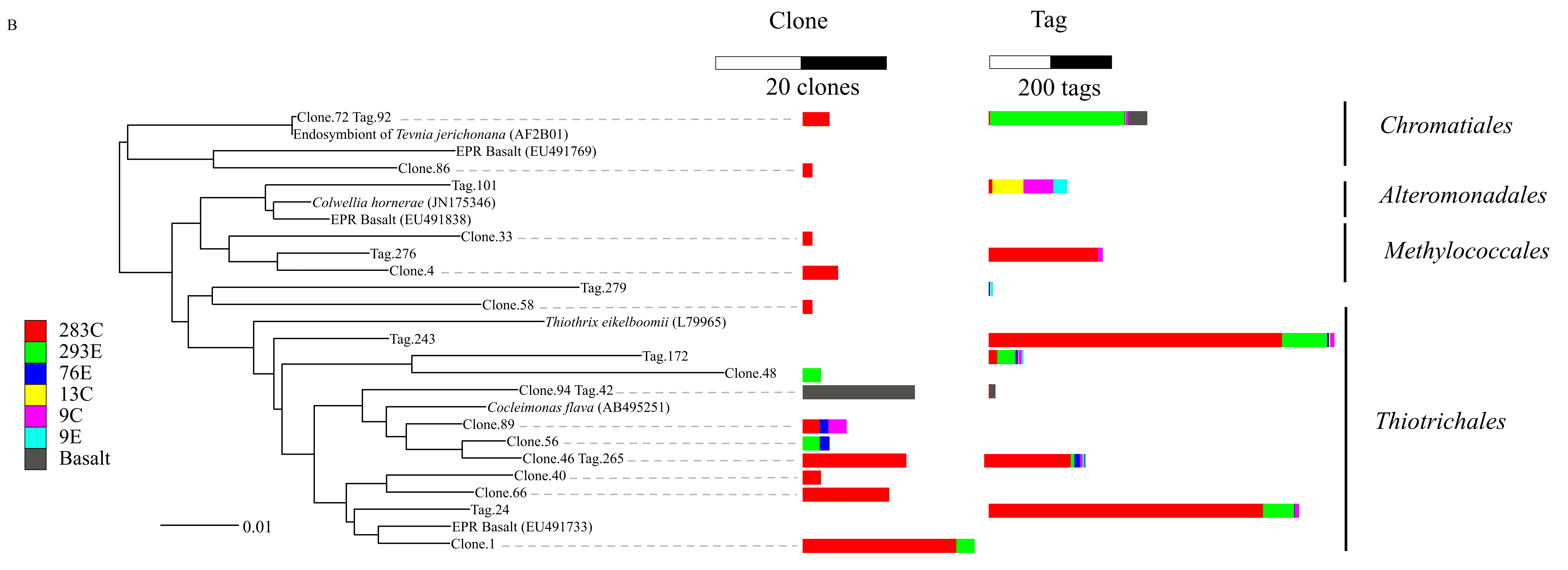

Supplementary Figure 4B. *Gammaproteobacteria* phylogenetic tree. Trees were constructed using the neighbor-joining algorithm from aligned, full-length clone sequences using the ARB software package. Tag sequences found at high abundances (>50 tags detected per sample), as well as those that were identical to a clone sequence or those that represent a unique lineage were inserted into trees using the parsimony insertion tool.
